# The GATA3-STEAP4 Axis Drives Inflammation by Promoting Th2 Differentiation in Allergic Rhinitis

**DOI:** 10.1007/s10753-025-02381-7

**Published:** 2025-12-20

**Authors:** Xiaoxu Ding, Hui Su, Tiancong Liu, Yu Chen, Zhao Gao, Ziwen Chang, Weiliang Bai

**Affiliations:** Department of Otolaryngology Head and Neck Surgery, Shengjing Hospital of China Medical University, Shenyang, China

**Keywords:** Allergic rhinitis, STEAP4, GATA3, Th2 differentiation, Inflammation

## Abstract

Allergic rhinitis (AR) is an inflammatory disease of the upper airway that primarily affects the nasal mucosa, with Th2 differentiation-driven inflammation as a key contributor. A bioinformatics analysis of dataset GSE52804 identified Six-transmembrane epithelial antigen of prostate 4 (STEAP4), a metalloreductase involved in inflammation regulation, as associated with AR progression, though its specific function remains unclear. Data obtained from nasal mucosal tissues from AR patients (*n* = 13) and an ovalbumin (OVA)-induced AR mouse model demonstrated a marked upregulation of STEAP4. Subsequent loss-of-function experiments revealed that STEAP4 knockdown reduced Th1/Th2 imbalance-mediated inflammation, alleviating allergic symptoms in OVA-treated mice. Further investigations involved the purification of naïve CD4^+^ T cells from healthy murine splenocytes and their Th2 polarization. Consistently, STEAP4 knockdown inhibited Th2 differentiation and the production of Th2-related cytokines in vitro. Additionally, Guanine adenine thymine adenine sequence-binding protein 3 (GATA3), a transcription factor essential for Th2 differentiation, was predicted to bind to the STEAP4 promoter. CHIP-PCR and dual-luciferase assays confirmed the transcriptional regulation of STEAP4 by GATA3. More importantly, STEAP4 knockdown rescued the promoting effects of GATA3 overexpression on Th2 differentiation. In conclusion, STEAP4 functions downstream of GATA3 to promote AR development by promoting Th2 differentiation-mediated inflammation, suggesting its potential as a target for AR treatment.

**Supplementary Information:**

The online version contains supplementary material available at 10.1007/s10753-025-02381-7.

## Introduction

Allergic rhinitis (AR) is an inflammatory condition triggered by the inhalation of allergens, resulting in immunoglobulin E (IgE)-mediated immune responses [1]. This condition predominantly affects the nasal mucosa and is characterized by symptoms including sneezing, nasal obstruction, and itchiness [2]. It is estimated that approximately 40% of the global population is affected by AR [3]. The persistent nature of this disorder presents substantial social and medical challenges [4]. The pathophysiology of allergic inflammation involves the abnormal differentiation of T helper (Th) cells, particularly Th2 cells, which secrete key cytokines such as IL-4, IL-5, and IL-13, thereby driving allergic immune responses [5–8]. Consequently, the inhibition of Th2 differentiation might serve as a promising strategy for the management of AR.

Six-transmembrane epithelial antigen of prostate 4 (STEAP4), also known as six transmembrane protein of the prostate 2 (STAMP2), belongs to the STEAP family and possesses metallo-reductase activity associated with copper and iron transport [9]. Initially identified in the adipose tissue, STEAP4 exhibits highly sensitivity to pro-inflammatory cytokines [5]. Numerous studies have demonstrated its involvement in various inflammation-related biological processes. In experimental autoimmune encephalomyelitis, STEAP4 has been identified as a key effector molecule in promoting Th17-mediated neuro-inflammatory pathogenesis [10]. Additionally, an upregulation of STEAP4 expression has been observed in the colon tissues of mice with colitis induced by Dextran Sulfate Sodium (DSS) treatment [11]. Notably, analysis of data from the GSE52804 dataset (https://www.ncbi.nlm.nih.gov/) reveals a significant increase in STEAP4 expression in the nasal mucosa of OVA-treated mice. However, the role of STEAP4 in modulating Th2 differentiation during allergic reactions remains largely unexplored.

The biological effects of STEAP4, as well as its implications in disease pathology, are influenced by various factors, including critical transcription factors. One important transcription factor Guanine adenine thymine adenine sequence-binding protein 3 (GATA3), is crucial for Th2 differentiation [12]. Significantly elevated GATA3 expression has been determined in the nasal mucosa of AR patients following allergen provocation [13]. Moreover, we found the downregulation of STEAP4 expression in CD4^+^ T cells following GATA3 knockdown in the GSE46333 dataset (https://www.ncbi.nlm.nih.gov/). Utilizing the JASPAR database (https://jaspar.elixir.no/), we predicted that GATA3 binds to the promoter region of STEAP4. Therefore, we hypothesized that STEAP4, as a transcriptional target of GATA3, influences Th2 differentiation in the context of AR.

To test this hypothesis, we collected nasal mucosal tissues from patients with AR and healthy individuals to examine the expression profile of STEAP4 within AR pathology. Next, we employed an OVA-induced AR mouse model and conducted loss-of-function experiments to assess the effects of STEAP4 on AR progression. Furthermore, we isolated naïve CD4^+^ T from murine splenocytes and cultured them under conditions of Th2 polarization, aiming to elucidate the role of STEAP4 in Th2 differentiation. We also verified the regulatory relationship between GATA3 and STEAP4. Our findings suggest that the GATA3/STEAP4 axis might represent a novel therapeutic target for AR treatment.

## Methods

### Bioinformatics Dataset Analysis

The GSE52804 dataset was retrieved from the NCBI GEO database (https://www.ncbi.nlm.nih.gov/geo/). Differentially expressed genes (DEGs) were identified according to the criteria of |Log2FC| > 1 and *p* < 0.01. Following identification, DEGs underwent gene ontology (GO)-biological process (BP) enrichment analysis to assess their functional implications.

## Clinical Samples

Patients with AR were recruited, and their nasal mucosal tissues were collected for the analysis of STEAP4 and GATA3 expression. Accordingly, control samples comprised nasal mucosal tissues obtained from patients undergoing nasal or plastic surgery.

## Construction of the Recombinant Lentiviral Vectors

For STEAP4 knockdown, a specific shRNA sequence targeting STEAP4 (GCGCAGTGCTGGTGCTGAAGT) was inserted into the lentiviral shuttle vector pLKO.1-EGFP-puro (Hunan Fenghui Biotechnology Co., Ltd, Changsha, Hunan, China). To achieve GATA3 overexpression, the coding sequences of GATA3 were inserted into the lentiviral shuttle vector pLVX-IRES-puro (Hunan Fenghui Biotechnology Co., Ltd, Changsha, Hunan, China). Subsequently, the shuttle vector, the packaging vector pSPAX2 (Hunan Fenghui Biotechnology Co., Ltd, Changsha, Hunan, China), and the envelope vector pMD2.G (Hunan Fenghui Biotechnology Co., Ltd, Changsha, Hunan, China) were co-transfected into HEK293T cells (iCell Bioscience Inc., Shanghai, China) using Lipofectamine 3000 (Invitrogen, Carlsbad, CA, USA). After 48 h of transfection, the supernatant containing viral particles was harvested and filtered through a 0.45-µm filter to obtain the recombinant lentiviral vectors (Lv-shSTEAP4 and Lv-GATA3). As a negative control for the Lv-shSTEAP4, the lentiviral vector carrying a non-targeting shRNA was utilized, while an empty lentiviral vector served as a negative control for Lv-GATA3. The maps of recombinant shuttle vectors are displayed in Figure S1.

## Animal Experiments

Female BALB/c mice aged 4 weeks were used to establish a mouse model of AR following a previously validated protocol [14]. Mice were injected intraperitoneally with 200 µL of normal saline containing 25 µg of ovalbumin (OVA) and 1 mg of aluminum hydroxide (AH) on days 0, 7, and 14. Subsequently, from days 21 to 27, 500 µg of OVA was given to mice *via* intranasal infusion per day. After OVA challenge on day 27, the number of sneezes and nose rubbing within 15 min were counted. On day 28, mice were euthanized for the collection of nasal mucosal tissues.

Additionally, to investigate the role of STEAP4 in the mouse model of AR, female BALB/c mice aged 4 weeks underwent identical treatment regimen involving OVA and AH on days 0, 7, and 14. On day 19, 8 µL of lysophosphatidylcholine (LPC) was instilled intranasally into mice. Following a 30 min interval, 20 µL (1 × 10^9^ TU/mL) of the lentiviral vector carrying the shRNA targeting STEAP4 (Lv-shSTEAP4) or lentiviral non-targeting shRNA (Lv-shNC) was administered intranasally into mice [15]. Mice were then challenged with 500 µg of OVA daily from days 21 to 27. On day 27, the frequency of sneezes and nose rubbing within 15 min post-OVA treatment were recorded. Mice were euthanized on day 28 to collect nasal mucosa tissues, spleens, blood, and nasal lavage fluid.

## Real-time PCR

The total RNA was extracted from nasal mucosal tissues and cells using TRIZOL reagent (BioTeke, Beijing, China). The RNA was subsequently reverse-transcribed into complementary DNA (cDNA), which was amplified using the specific primers for PCR analysis. The cycle threshold (Ct) value was normalized to β-actin to calculate the relative mRNA expression of genes according to the 2 ^−∆Ct^ (clinical samples) or 2 ^−∆∆Ct^ method (tissues or cell samples from mice). For tissues or cell samples from mice, the mRNA level of genes in the control group was set as 1. Specifically, the mean values of the Ct (target gene) and Ct (internal control) in the control group were calculated, respectively. Subsequently, ∆Ct = mean Ct (target gene) - mean Ct (internal control) and ∆∆Ct = ∆Ct - ∆Ct (the control group) were calculated, and the relative mRNA expression of genes was defined as 2 ^−∆∆Ct^. Since that ∆∆Ct (the control group) is equal to its own subtraction, and the result is 0. Therefore, 2 ^−∆∆Ct^ (the control group) = 1. Sequences of specific primers for genes are listed in Table S1.

### Western Blot

The total protein was extracted from nasal mucosal tissues and cells using RIPA lysis buffer supplemented with a Common Protease Inhibitor Cocktail (Proteintech Group Inc., Wuhan, Hubei, China). BCA assays were performed to determine the protein concentrations. Protein samples were run through SDS-PAGE, and the separated protein were transferred to PVDF membranes (Thermo Fisher Scientific, Waltham, MA, USA). Skim milk solution was used to block the non-specific binding sites on membranes. The blots were incubated with primary and secondary antibodies followed by substrate detection using the ECL reagent (Proteintech Group Inc., Wuhan, Hubei, China). STEAP4 antibody (1:1000, Proteintech Group Inc., Wuhan, Hubei, China) was used to incubate membranes under the condition of 4 °C overnight, and its corresponding secondary antibody goat anti-rabbit IgG-HRP (1:10000, Proteintech Group Inc., Wuhan, Hubei, China) was used to incubate membranes under the condition of 37 °C for 40 min. β-actin (1:20000, Proteintech Group Inc., Wuhan, Hubei, China) was used as the internal control, along with its corresponding secondary antibody goat anti-mouse IgG-HRP (1:10000, Proteintech Group Inc., Wuhan, Hubei, China). In the western blot analysis, all biological replicates were processed independently. For each biological replicate, the band density of each experimental group was normalized to the internal control. The protein expression level for each group was then calculated as the fold change relative to the control group, with the control group assigned a value of 1 relative to itself. Consequently, across all biological replicates, the expression value of the control group was consistently defined as 1.

## Immunofluorescence

Fixed tissues underwent a gradient ethanol dehydration process followed by embedding in paraffin. The paraffin-embedded tissues were then sectioned into 5 μm thick slices, which were dewaxed using xylene and subsequently rehydrated through a series of ethanol solutions. Antigen retrieval was carried out in an antigen repair solution under high temperature at low heat for 10 min. Tissue sections were blocked with a 1% BSA solution prior to incubation with primary and secondary antibodies. Following nuclear staining with DAPI (Aladdin, Shanghai, China), the tissues were mounted with an anti-fluorescence quencher (Solarbio, Beijing, China) and observed under a microscope (Olympus, Tokyo, Japan). The STEAP4 antibody (Proteintech Group Inc., Wuhan, Hubei, China) or CDH26 antibody (Affinity, Liyang, Jiangsu, China) was diluted at a ratio of 1:100 and used to incubate sections under the condition of 4 °C overnight. Goat anti-rabbit IgG-Cy3 (1:200, Proteintech Group Inc., Wuhan, Hubei, China) was used as its corresponding secondary antibodies at room temperature for 60 min. For quantitative analysis, STEAP4-positive cells were manually counted.

## Hematoxylin and Eosin (H&E) Staining

Fixed tissues were flushed with running water and then dehydrated using a gradient ethanol solution. They were subsequently embedded in paraffin and cut into 5 μm thick sections. The sections were dewaxed in xylene and rehydrated with a gradient ethanol solution. Following this, the sections were stained with hematoxylin (Solarbio, Beijing, China) for 5 min and differentiated using a 1% hydrochloric acid alcohol solution. After being rinsed with running water and immersed in distilled water, the sections were stained with eosin Y (Sangon Biotech Co., Shanghai, China) for 3 min. Finally, the sections were dehydrated with a gradient ethanol solution, cleared in xylene, mounted with neutral gum, and imaged under a microscope (Olympus, Tokyo, Japan).

### Periodic Acid-Schiff (PAS) Staining

PAS staining was performed using a PAS staining solution (Leagene, Beijing, China). Briefly, the dehydrated tissue was embedded in paraffin and sliced into 5-µm sections. The sections were then placed in xylene for dewaxing, followed by rehydration in a gradient of ethanol. Next, the sections were immersed in a periodic acid solution within a humid box for 10 min. After a 5-min immersion in distilled water, the sections were stained with Schiff solution in the humid box for 15 min. After rinsing in water for 5 min, hematoxylin was applied as a counterstain for 2 min, followed by a 10 min-rinse in tap water to restore the blue color. Sections were then rehydrated with a gradient ethanol solution and cleared in xylene. Finally, neutral gum was used to mount the sections, which were examined under a microscope (Olympus, Tokyo, Japan).

### Sirius Red Staining

Sirius red staining was performed using an Eosinophil Stain Kit (Solarbio, Beijing, China). In detail, the tissues were dehydrated using a gradient ethanol solution and then embedded in paraffin. They were prepared into 5-µm sections, which underwent dewaxing in a xylene solution followed by rehydration in a gradient of ethanol. Next, the sections were stained with Sirius red solution for 24 h. Following that, sections were rinsed with distilled water for 5 min and then dehydrated again with a gradient of ethanol. After being cleared in xylene and mounted with neutral gum, the sections were viewed under a microscope (Olympus, Tokyo, Japan).

### Diff-quik Staining

The nasal lavage fluid (NALF) of mice was collected and centrifuged to obtain the cell pellets, which were resuspended in 0.5 mL of PBS. A small amount of solution was pipetted onto the hemocytometer for counting the number of white blood cells under a microscope (Olympus, Tokyo, Japan). Additionally, 10 µL of solution was dripped onto clean slides and made into cell smears to count the number of different immune cells, including lymphocytes, eosinophils, neutrophils, and macrophages. Slides were successively stained with Diff-Quik Ⅰ dye (Leagene, Beijing, China) for 5 ~ 10 s and Diff-Quik Ⅱ dye (Leagene, Beijing, China) for 5 ~ 10 s. After being flushed with distilled water, slides were observed under a microscope (Olympus, Tokyo, Japan) to count cell numbers.

### ELISA

Serum levels of IgG1, IgG2a, and IgE were measured using commercially available kits to determine the OVA-specific antibody production. Mouse OVA sIgG1 ELISA Kit, Mouse OVA sIgG2a ELISA Kit, and Mouse OVA sIgE ELISA Kit were purchased from Fine Test (Wuhan, Hubei, China). Serum histamine was measured using a commercially available kit to indicate the concentration of this critical mediator in the development of AR. HIS (Histamine) ELISA Kit was purchased from Fine Test (Wuhan, Hubei, China). Furthermore, the concentrations of inflammatory cytokines TSLP, IFN-γ, IL-4, IL-5, and IL-13 in NALF were measured using commercially available kits. Mouse TSLP ELISA Kit, Mouse IFN-gamma ELISA Kit, Mouse IL-4 ELISA Kit, Mouse IL-5 ELISA Kit, and Mouse IL-13 ELISA Kit were purchased from MULTI SCIENCES (Hangzhou, Zhejiang, China).

### Isolation of Splenic Mononuclear Cells and Cell Treatment

Spleens were collected from mice with OVA challenge or their controls under aseptic conditions and cut into small pieces after splenic capsules were removed. Spleen mononuclear cells were isolated using the Mouse Spleen Mononuclear Cell Isolation Kit (Solarbio, Beijing, China), referring to a previous research [16]. Briefly, tissue pieces were placed on a screen and ground using sterile tweezers. After grinding completely, the screens were washed with the whole blood and tissue diluent to collect cell suspension, which was filtered through a cell strainer. The cell suspension was added to a centrifuge tube containing an equal amount of isolation fluid. After centrifugation, the second opalescent mononuclear cells were moved into another centrifuge tube and washed with the washing liquid. Centrifugation was performed to discard the supernatant, and the cell pellets were collected for subsequent use.

According to a previous report [17], cells were co-administered with 75 ng/mL of Phorbol12-myristate13-acetate (PMA), 1 µg/mL of Ionomycin, and 1 µL/mL of BD GolgiPlug containing Brefeldin A (Macklin, Shanghai, China) for 4 h.

### Isolation of Splenic Naïve CD4^+^ T Cells and Cell Differentiation

Spleens were harvested from healthy 4-week-old female BALB/c mice to isolate splenocytes. Naïve CD4^+^ T cells were purified using an immunomagnetic bead-based sorting method, specifically employing the Mouse naïve CD4^+^ T Cell Isolation Kit (Miltenyi Biotec, Bergisch Gladbach, Germany). Briefly, cells were resuspended in buffer solution and incubated with Biotin-Antibody Cocktail at 2 ~ 8 °C for 5 min. The buffer solution and Anti-Biotin MicroBeads were added to treated cells at 2 ~ 8 °C for another 10 min. The LS column was placed in the magnetic field of a separator and washed with the buffer solution. Cell suspension was loaded into the column, and unlabeled cells passing through the column were collected, namely enriched CD4^+^ T cells.

Purified naïve CD4^+^ T cells were induced to differentiate into Th2 cells according to the previously published literature with some modifications [18–20]. In brief, CD4^+^ T cells were activated with 5 µg/mL of anti-CD3 and 2 µg/mL of anti-CD28 (BioXcell, West Lebanon, NH, USA) for 5 days. Then, 20 ng/mL of IL-4 (MCE, Birmingham, NJ, USA) and 10 µg/mL of anti-IFN-γ (Thermo Fisher Scientific, Waltham, MA, USA) were added to treat cells for 72 h to induce Th2 differentiation.

For Th1, Th17, and Treg differentiation, naïve CD4^+^ T cells were stimulated with plate-bound anti-CD3 (5 µg/mL) and anti-CD28 (2 µg/mL) in the presence of different polarization cytokines for 5 days. For Th1 differentiation, IL-12 (10 ng/mL, MCE, Birmingham, NJ, USA) and anti-IL-4 (10 µg/mL) were added; for Th17 differentiation, TGF-β (2 ng/mL, MCE, Birmingham, NJ, USA), IL-6 (30 ng/mL, MCE, Birmingham, NJ, USA), IL-1β (10 ng/mL, MCE, Birmingham, NJ, USA), IL-23 (20 ng/mL, MCE, Birmingham, NJ, USA), anti-IFN-γ (10 µg/mL, Thermo Fisher Scientific, Waltham, MA, USA), and anti-IL-4 (10 µg/mL) were added; for Treg differentiation, TGF-β (5 ng/mL), IL-2 (10 ng/mL), anti-IFN-γ (5 µg/mL), and anti-IL-4 (5 µg/mL) were added [20].

### Lentiviral Infection

For gene knockdown or overexpression in CD4^+^ T cells, the liquid containing lentivirus particles and Polybrene were used to incubate cells at room temperature for 15 ~ 30 min (MOI = 150). Cells and the liquid were then transferred to culture plates and supplemented with the culture medium. After 48 h of infection, CD4^+^ T cells were induced to differentiate into Th2 cells.

### Flow Cytometry

The antibodies against APC-Anti-Mouse CD4 (MULTI SCIENCES, Hangzhou, Zhejiang, China) and FITC-Anti-Mouse CD25 (Elabscience, Wuhan, Hubei, China) were added to the cell suspension to incubate cells at 4 °C for 30 min in the dark. After being treated with Fixation/Permeabilization solution (MULTI SCIENCES, Hangzhou, Zhejiang, China), cells were incubated with PE-anti-mouse IFN-γ antibody (Biolegend, San Diego, CA, USA), PE-Anti-Mouse IL-4 antibody (Proteintech Group Inc., Wuhan, Hubei, China), PE-Anti-Mouse IL-17 A antibody (Elabscience, Wuhan, Hubei, China), or PE-Anti-Mouse FOXP3 antibody (Elabscience, Wuhan, Hubei, China) at 4 °C for 30 min in the dark. Cells to be tested were resuspended in 500 µL of the buffer solution, which was subjected to flow cytometry analysis (Agilent, Santa Clara, CA, USA).

### Dual-luciferase Assay

HEK293T cells were cultured in Dulbecco’s modified eagle medium (Servicebio, Wuhan, Hubei, China) supplemented with 10% fetal bovine serum in an incubator with 5% CO_2_ at 37 °C. The binding sites of GATA3 within the STEAP4 promoter were predicted using the JASPAR website (https://jaspar.elixir.no/). A dual-luciferase assay was performed to verify these predictions. The sequences of the STEAP4 promoter were inserted into the pGL3-basic luciferase reporter vector. Further, the plasmid expressing GATA3 was constructed, and the corresponding empty vector was used as the negative control. Next, HEK293T cells were co-transfected with the GATA3-overexpressing vector (or the empty vector), pGL3-basic-STEAP4 promoter, and the pRL-TK vector. The relative luciferase activity was detected by Dual Luciferase Reporter Gene Assay Kit (Keygentec, Nanjing, Jiangsu, China) using a microplate reader (TECAN, Mannedorf, Switzerland).

### ChIP-PCR

A ChIP assay was performed using the ChIP Assay Kit (Beyotime Biotechnology, Shanghai, China). In brief, CD4^+^ T cells were treated with formaldehyde solution at a final concentration of 1% at 37 °C for 10 min to cross-link the target protein with the corresponding genomic DNA. Glycine Solution (10X) was added to treat cells at room temperature for 5 min. Cells were washed with pre-cooled PBS containing 1 mM PMSF and centrifuged to collect the cell pellet, which was resuspended in the SDS Lysis Buffer containing 1 mM PMSF in an ice bath for 10 min to lyse cells. Genomic DNA fragments were sheared to 200 ~ 500 bp fragments using ultrasonic processing in an ice-bath. The samples were centrifuged to obtain the supernatant, which was diluted with ChIP Dilution Buffer containing 1 mM PMSF. Some part of the diluted supernatant was used as the “Input” to perform PCR experiments. The remaining diluted supernatant was treated with Protein A + G Agarose/Salmon Sperm DNA at 4 °C for 30 min. After centrifugation, the supernatant was collected and incubated with IgG antibody or GATA3 antibody at 4 °C overnight. Protein A + G Agarose/Salmon Sperm DNA was added to precipitate the complex identified by the primary antibody at 4 °C for 60 min. The supernatant was removed after centrifugation, and the sediment was washed with Low Salt Immune Complex Wash Buffer, High Salt Immune Complex Wash Buffer, LiCl Immune Complex Wash Buffer, and TE Buffer, successively. The ChIP products were analyzed by PCR detection.

### Statistical Analysis

Data were displayed in the form of mean ± SD and analyzed by Graphpad 9. Unpaired t test, Welch’s t test, or Mann-Whitney test was used to compare the mean value between two groups. One-way ANOVA or Brown-Forsythe and Welch ANOVA tests was used to compare the mean value among three or more than three groups. Two-way ANOVA was used to compare the mean value with two factors. Additionally, Pearson correlation analysis was performed to assess the relationship between mRNA expression levels of STEAP4 and GATA3.

## Results

### Higher STEAP4 Expression Might be Associated with AR

**Fig. 1:**
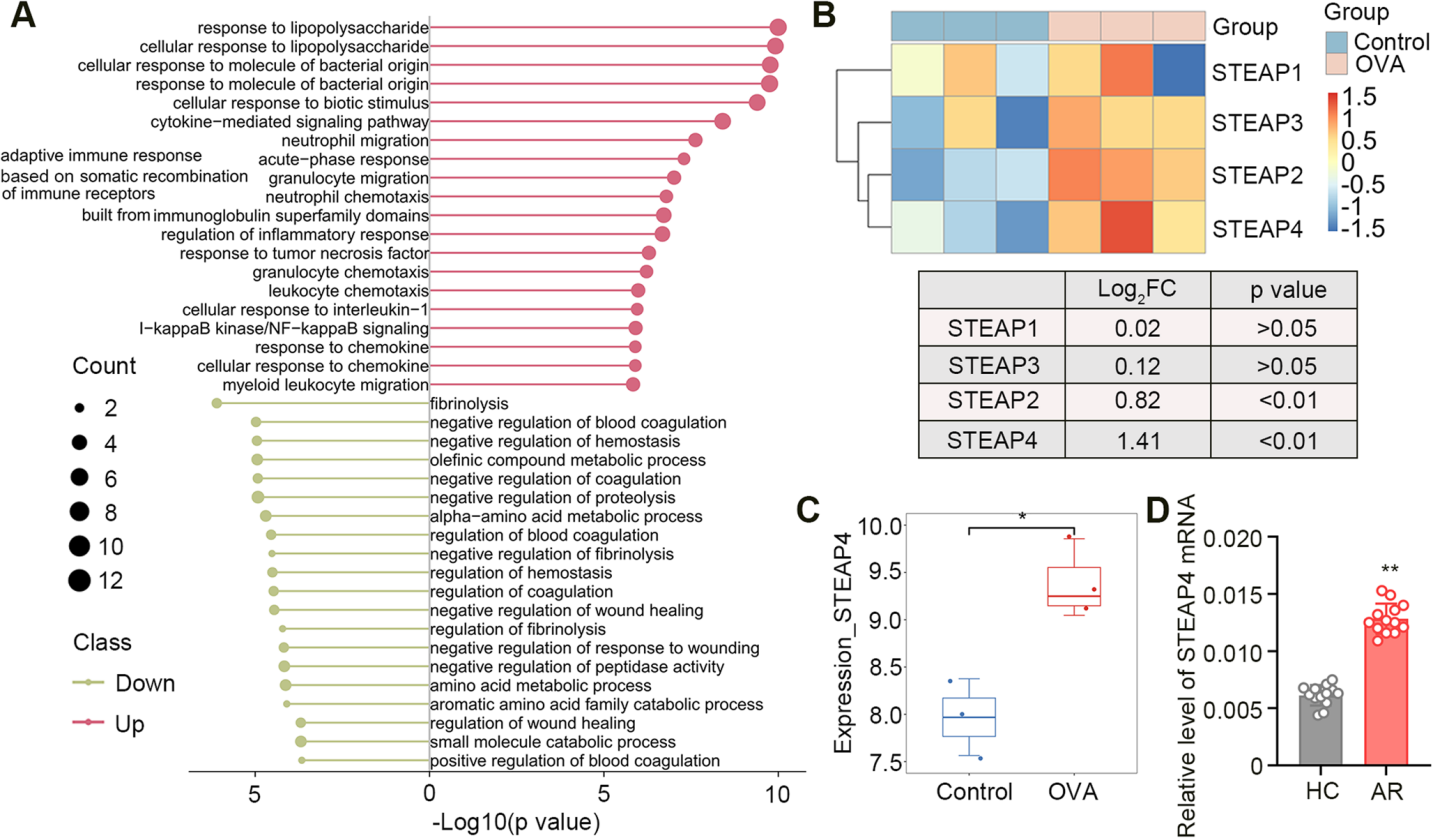
Increased STEAP4 expression was found in AR samples. (**A**) The top 20 of GO-BP terms enriched by upregulated and downregulated DEGs. (**B**) The heat map and the expression profiles of the STEAP family genes in GSE52804. (**C**) The STEAP4 expression in GSE52804 (*n* = 3). (**D**) The mRNA expression of STEAP4 in nasal mucous tissues of patients (*n* = 13). For Fig. 1D, the AR group vs. the HC group, **p < 0.01. Data were presented as the mean ± SD and analyzed by unpaired t test. Each experiment was independently reproduced

Given the high incidence and treatment costs of AR, it is essential to explore the molecular targets associated with AR to provide new insights into its therapy. Therefore, gene expression microarray data were retrieved from the NCBI GEO database (accession number: GSE52804) and used for bioinformatics analysis aimed at identifying candidate genes related to AR. The distribution of DEGs regulated by OVA challenge in the mouse nasal mucosa is presented in the volcano plot (Fig. S2). A heat map displayed the expression profiles of these DEGs (Fig. S3). Next, upregulated and downregulated DEGs were annotated into GO enrichment terms of BP, respectively. We found that multiple BP pathways enriched by upregulated DEGs were more associated with immune and inflammatory response than those enriched by downregulated DEGs (Fig. 1A). Interestingly, we noticed that the STEAP family, which regulates iron and copper homeostasis, may be closely related to Th2 immune response [21, 22]. We thus pulled the expression patterns of STEAP family genes in GSE52804 and depicted the corresponding heat map (Fig. 1B). Among them, STEAP4 is the only one that is differentially expressed (Fig. 1C). Based on these findings, we speculated that STEAP4 might play an important role in the development of AR. To further investigate, we collected nasal mucous tissues from clinical patients to determine the STEAP4 expression. The results from real-time PCR showed that STEAP4 was highly expressed in the nasal mucous tissues of patients with AR (Fig. 1D). These findings indicated that elevated expression of STEAP4 might be associated with the pathophysiology of AR.

### STEAP4 Was Highly Expressed in Nasal Mucous Tissues of OVA-treated Mice

**Fig. 2:**
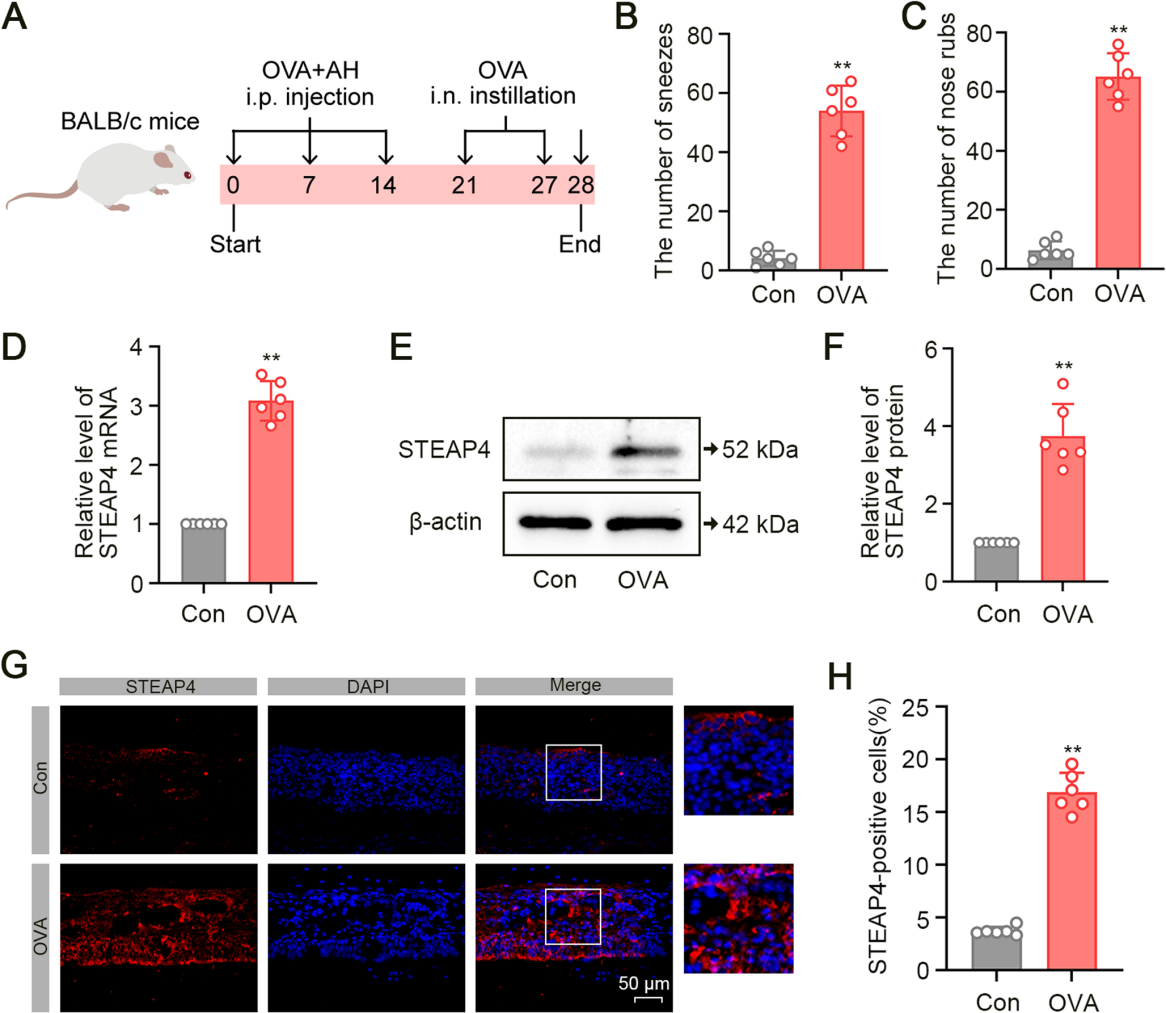
STEAP4 was highly expressed in OVA-induced mice with AR. (**A**) The timeline for the induction of the mouse model of AR. (**B**) The number of sneezes of mice (*n* = 6). (**C**) The number of nose rubs of mice (*n* = 6). (**D**) The mRNA expression of STEAP4 in nasal mucous tissues of mice (*n* = 6). (**E**) The protein expression of STEAP4 in nasal mucous tissues of mice. (**F**) Quantitative analysis of the STEAP4 expression in Fig. 2E (*n* = 6). (**G**) Representative images of immunofluorescence staining for the STEAP4 location in nasal mucous tissues of mice. (**H**) Quantitative analysis of the STEAP4 expression in Fig. 2G (*n* = 6). For Fig. 2B, D and F, and 2H, the OVA group vs. the Con group, **p < 0.01. Data were presented as the mean ± SD and analyzed by unpaired t test, Welch’s t test, or Mann-Whitney test. Each experiment was independently reproduced

Next, a mouse model of AR induced by OVA challenge was constructed for further exploration. The treatment timeline is illustrated in Fig. 2A. Sneezing and itching nose are well-established symptoms of AR [23]. Therefore, we recorded the number of sneezes and nose rubbing in mice over a 5-min observation period. The results showed that mice with OVA challenge exhibited the obvious AR symptoms, as evidenced by a marked increase in both sneezes and nose rubbing compared to their controls (Fig. 2B and C). Moreover, we found the increased STEAP4 mRNA expression in nasal mucous tissues of mice with AR (Fig. 2D). Correspondingly, the STEAP4 protein expression exhibited a similar trend (Fig. 2E and F). Representative images of immunofluorescence staining located the increased STEAP4 expression in the nasal mucous tissue of OVA-challenged mice (Fig. 2G and H). These results indicated that highly expressed STEAP4 was related to AR.

#### STEAP4 Knockdown Alleviated the OVA-induced AR Symptoms

**Fig. 3:**
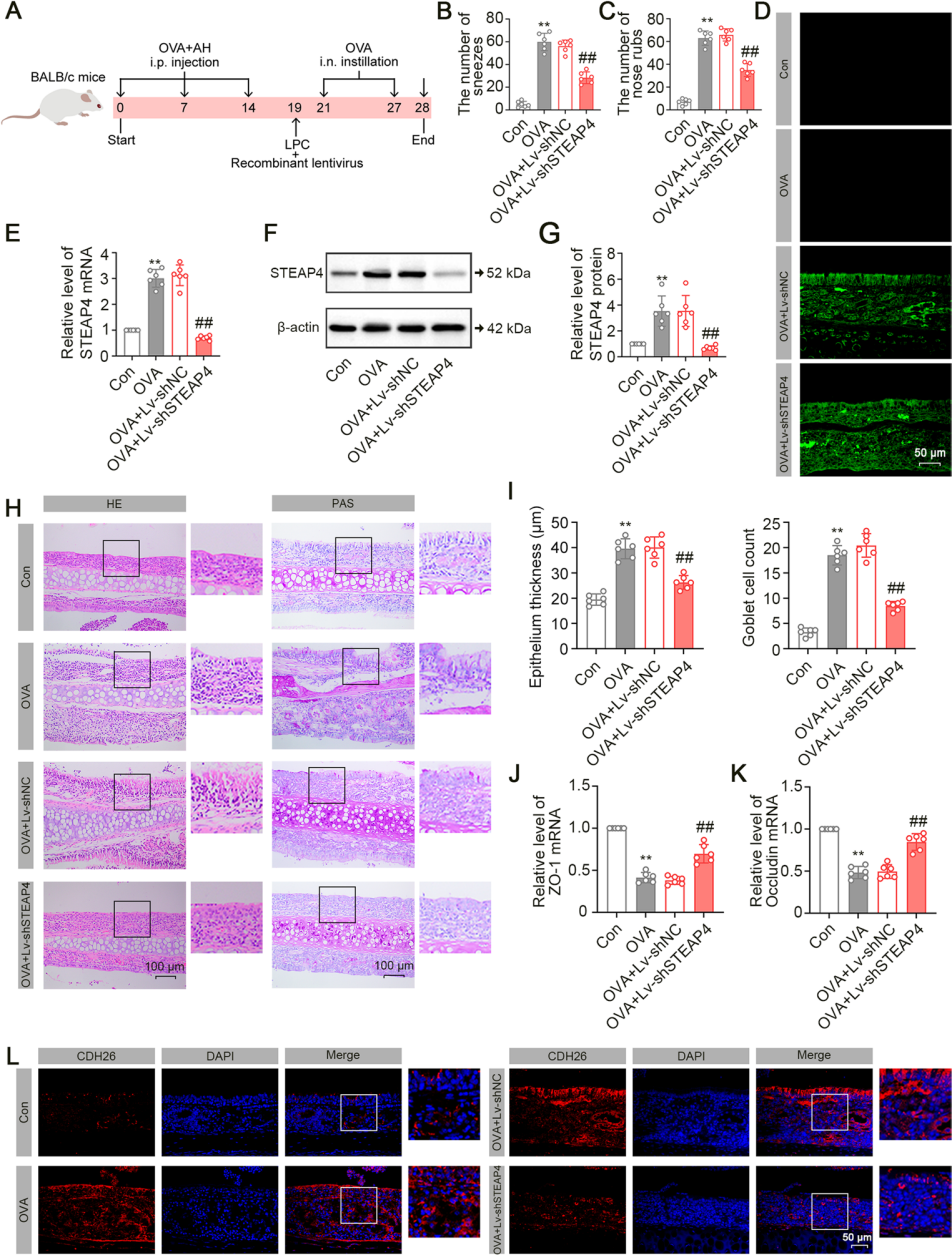
STEAP4 knockdown alleviated OVA-induced AR symptoms in mice. (**A**) The timeline for lentiviral administration in OVA-challenged mice. (**B**) The number of sneezes of mice (*n* = 6). (**C**) The number of nose rubs of mice (*n* = 6). (**D**) Representative images of GFP fluorescence for determination of lentiviral infection efficiency in nasal mucous tissues of mice. (**E**) The mRNA expression of STEAP4 in nasal mucous tissues of mice (*n* = 6). (**F**) The protein expression of STEAP4 in nasal mucous tissues of mice. (**G**) Quantitative analysis of the STEAP4 expression in Fig. 3F (*n* = 6). (**H**) Representative images of H&E staining for pathological changes and PAS staining for goblet cell distribution in nasal mucous tissues of mice. (**I**) Quantitative analysis of epithelial thickness and goblet cell count in Fig. 3H (*n* = 6). (**J**) The mRNA expression of ZO-1 in nasal mucous tissues of mice (*n* = 6). (**K**) The mRNA expression of Occludin in nasal mucous tissues of mice (*n* = 6). (**L**) Representative images of immunofluorescence staining for the CDH26 location in nasal mucous tissues of mice. For Fig. 3B, C, E, G, I and K, the OVA group vs. the Con group, **p < 0.01; the OVA + Lv-shSTEAP4 vs. the OVA + Lv-shNC group, ##*p* < 0.01. Data were presented as the mean ± SD and analyzed by one-way ANOVA or Brown-Forsythe and Welch ANOVA tests. Each experiment was independently reproduced

To explore the role of STEAP4 in the development of AR, mice were challenged with OVA for AR induction and administered with the recombinant lentivirus for STEAP4 knockdown in vivo. The experimental timeline was illustrated in Fig. 3A. Following STEAP4 knockdown, we observed a significant reduction in both the frequency of sneezes and nose rubbing of OVA-challenged mice (Fig. 3B and C). The knockdown efficiency of the recombinant lentivirus was visualized by GFP expression. Microscopy images showed the obvious GFP fluorescence in the nasal mucous tissues of OVA-challenged mice treated with Lv-shNC and Lv-shSTEAP4, but fluorescence was absent in the nasal mucosa tissues of mice from both the control and OVA groups (Fig. 3D). Subsequently, OVA-triggered increase in the STEAP4 mRNA and protein expression in nasal mucous tissues of mice were reduced by STEAP4 knockdown (Fig. 3E and G), suggesting the successful gene knockdown. Histological examinations using H&E staining, along with corresponding quantitative analysis, revealed an increased thickness of nasal mucosa in OVA-challenged mice; however, these morphological abnormality were diminished by STEAP4 knockdown. Further, PAS staining was performed to indicate goblet cells in nasal mucous tissues [24]. Goblet cell hyperplasia was found in mice challenged with OVA. STEAP4 knockdown inhibited the abnormal increase of goblet cells (Fig. 3H and I). We also assessed the expression of tight junction proteins ZO-1 and Occludin, which are essential for the maintenance of nasal mucosal barrier integrity [25]. OVA challenge inhibited the mRNA expression of ZO-1 and Occludin in nasal mucosal tissues of mice, suggesting the disruption of barrier integrity (Fig. 3J and K). Moreover, CDH26 was associated with epithelial cell dysfunction, and its knockdown alleviated AR-induced inflammatory response [26, 27]. Representative images of immunofluorescence staining showed that OVA challenge elevated the CDH26 expression in the nasal mucosal tissues of mice (Fig. 3L). However, STEAP4 knockdown reversed these trends, sustaining the epithelial barrier integrity under the AR condition (Fig. 3L). These results indicated that STEAP4 knockdown alleviated OVA-induced pathological features in mice.

#### STEAP4 Knockdown Inhibited OVA-induced Inflammatory Response

**Fig. 4:**
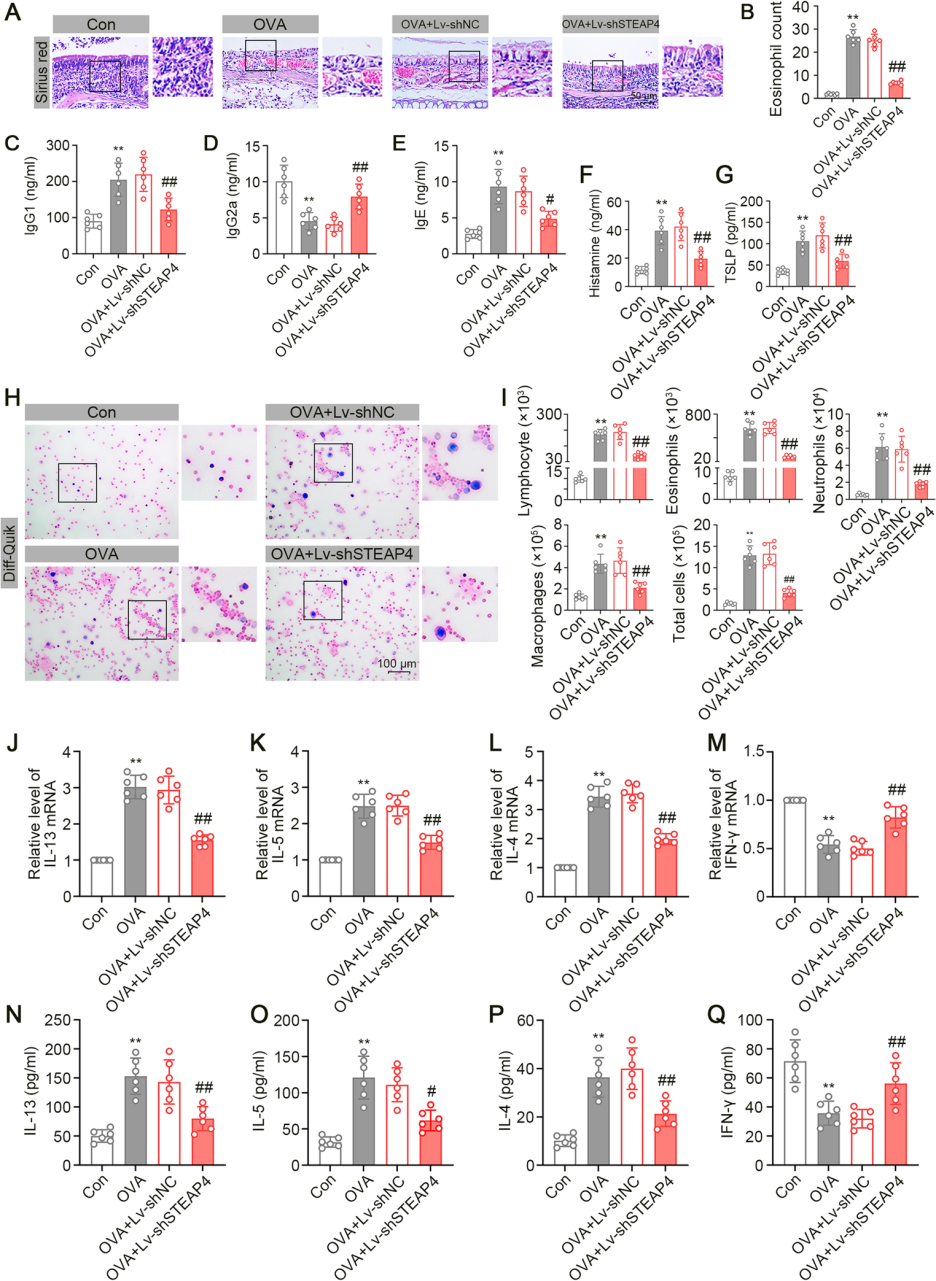
STEAP4 knockdown relieved Th2 immune response and inflammatory infiltrate. (**A**) Representative images of Sirius Red staining for eosinophils in nasal mucous tissues of mice. (**B**) Quantitative analysis of eosinophil count in Fig. 4A (*n* = 6). (**C**) The IgG1 concentration in serum of mice (*n* = 6). (**D**) The IgG2a concentration in serum of mice (*n* = 6). (**E**) The IgE concentration in serum of mice (*n* = 6). (**F**) The histamine concentration in serum of mice (*n* = 6). (**G**) The TSLP concentration in NALF of mice (*n* = 6). (**H**) Representative images of Diff-Quik staining of NALF. (**I**) The number of lymphocytes, eosinophils, neutrophils, macrophages, total cells in NALF of mice (*n* = 6). (**J**) The mRNA expression of IL-13 in nasal mucous tissues of mice (*n* = 6). (**K**) The mRNA expression of IL-5 in nasal mucous tissues of mice (*n* = 6). (**L**) The mRNA expression of IL-4 in nasal mucous tissues of mice (*n* = 6). (**M**) The mRNA expression of IFN-γ in nasal mucous tissues of mice (*n* = 6). (**N**) The concentration of IL-13 in NALF of mice (*n* = 6). (**O**) The concentration of IL-5 in NALF of mice (*n* = 6). (**P**) The concentration of IL-4 in NALF of mice (*n* = 6). (**Q**) The concentration of IFN-γ in NALF of mice (*n* = 6). For Fig. 4B, G, I and Q, the OVA group vs. the Con group, **p < 0.01; the OVA + Lv-shSTEAP4 vs. the OVA + Lv-shNC group, #*p* < 0.05, ##*p* < 0.01. Data were presented as the mean ± SD and analyzed by one-way ANOVA or Brown-Forsythe and Welch ANOVA tests. Each experiment was independently reproduced

Next, Sirius Red staining revealed an increase in eosinophils in the nasal mucosal tissue of AR-like mice, which was reversed by STEAP4 knockdown (Fig. 4A and B). Allergic inflammatory responses were assessed by the measurement of OVA-specific IgG1, IgG2a, and IgE concentrations in the serum [28]. We observed that exposure to OVA allergen resulted in the increased concentrations of IgG1 and IgE, while the concentration of IgG2a decreased. However, STEAP4 knockdown inhibited the allergic inflammatory responses induced by OVA (Fig. 4C and E). OVA administration led to an increase in the serum histamine level, but STEAP4 knockdown inhibited the serum histamine production in OVA-treated mice (Fig. 4F). The concentration of pro-allergic cytokine TSLP was found to be elevated in nasal mucous tissues of mice with AR, with the level decreasing upon STEAP4 knockdown (Fig. 4G). Next, the NALF was subjected to Diff-Quik staining to quantify inflammatory cell populations. The results showed that mice with OVA administration had more severe inflammatory cell infiltration compared to controls, as evidenced by an increased number of lymphocytes, eosinophils, neutrophils, macrophages, and the total cells. Notably, STEAP4 knockdown relieved inflammatory cell infiltration caused by OVA administration (Fig. 4H and I). Moreover, an imbalance of Th1/Th2 response is recognized as a common contributor to allergic reactions [29]. Based on that, the concentrations of Th1- and Th2-related pro-inflammatory cytokines and their mRNA expression were detected by ELISA kits and real-time PCR, respectively. OVA challenge inhibited the concentration and mRNA expression of Th1-related inflammatory cytokines (IFN-γ), while it increased the concentrations and mRNA expression of Th2-related inflammatory cytokines (IL-4, IL-5, and IL-13) (Fig. 4J and Q). These results indicated that STEAP4 knockdown inhibited inflammatory response in mice with AR.

#### STEAP4 Knockdown Modulated the Th1/Th2 Imbalance

**Fig. 5:**
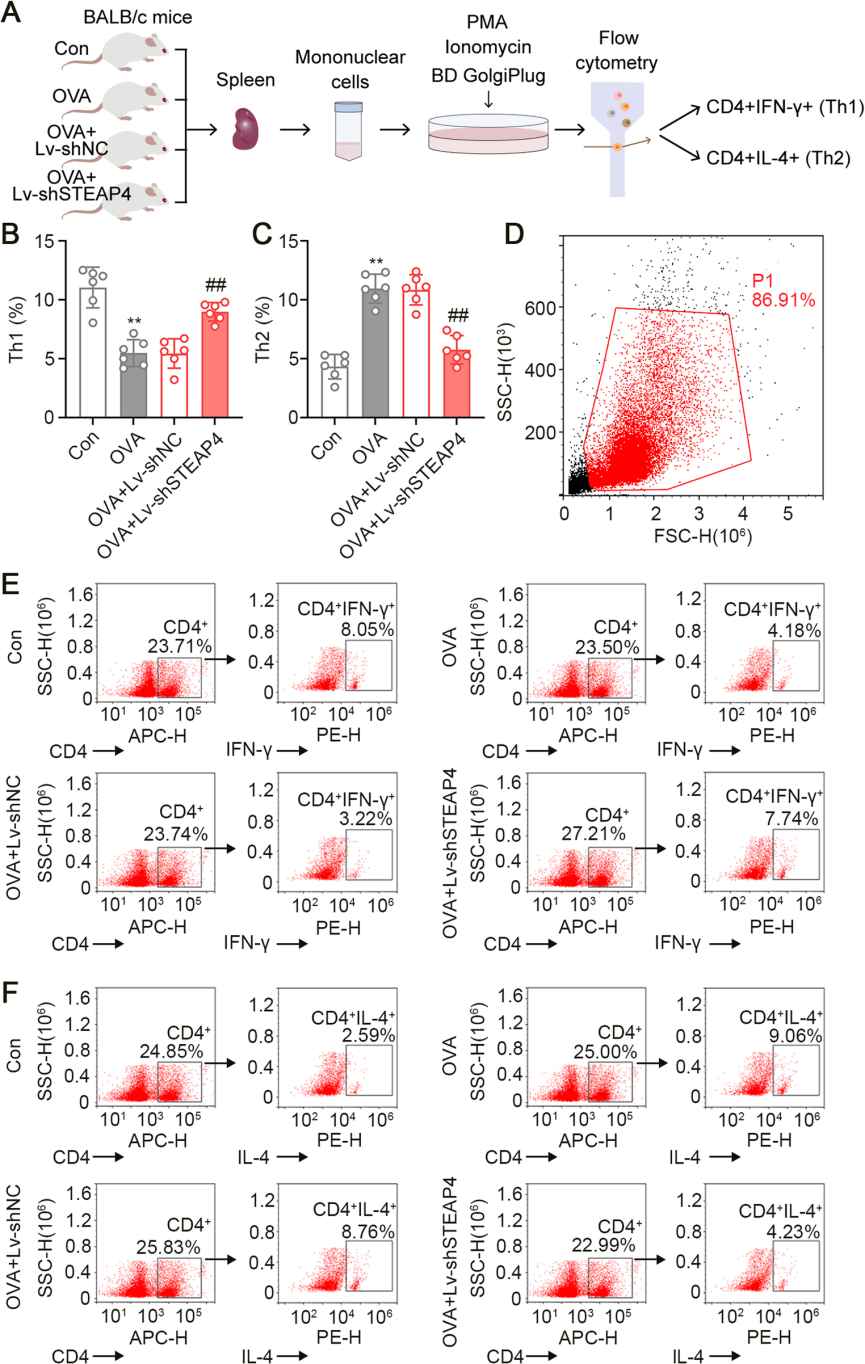
STEAP4 knockdown restored the Th1/Th2 imbalance induced by AR pathological condition. (**A**) The schematic diagram of splenocyte isolation and cell treatment. (**B**) The percentage of Th1 cells in splenocytes of mice (*n* = 6). (**C**) The percentage of Th2 cells in splenocytes of mice (*n* = 6). (**D**) The gating strategy for identifying Th1 and Th2 cells. (**E**) Scatter plots for the percentage of Th1 cells in splenocytes of mice. (**F**) Scatter plots for the percentage of Th2 cells in splenocytes of mice. For Fig. 5B and C, the OVA group vs. the Con group, **p < 0.01; the OVA + Lv-shSTEAP4 vs. the OVA + Lv-shNC group, ##*p* < 0.01. Data were presented as the mean ± SD and analyzed by one-way ANOVA. Each experiment was independently reproduced

We then isolated splenic mononuclear cells and treated them with PMA, Ionomycin, and BD GolgiPlug containing Brefeldin A to stimulate cytokine secretion. We determined the percentages of Th1 and Th2 cells by measuring their specific cytokines using flow cytometry. A schematic diagram of the isolation and treatment protocol for splenic mononuclear cells is illustrated in Fig. 5A. Similar to the observed trend in pro-inflammatory cytokines in vivo, OVA challenge significantly decreased the percentage of Th1 cells while increasing the percentage of Th2 cells. In contrast, STEAP4 knockdown resulted in an entirely opposite trend in mice with AR (Fig. 5B and F). These results showed that STEAP4 knockdown restored the Th1/Th2 imbalance.

### STEAP4 Knockdown Suppressed Th2 Cell Differentiation

**Fig. 6:**
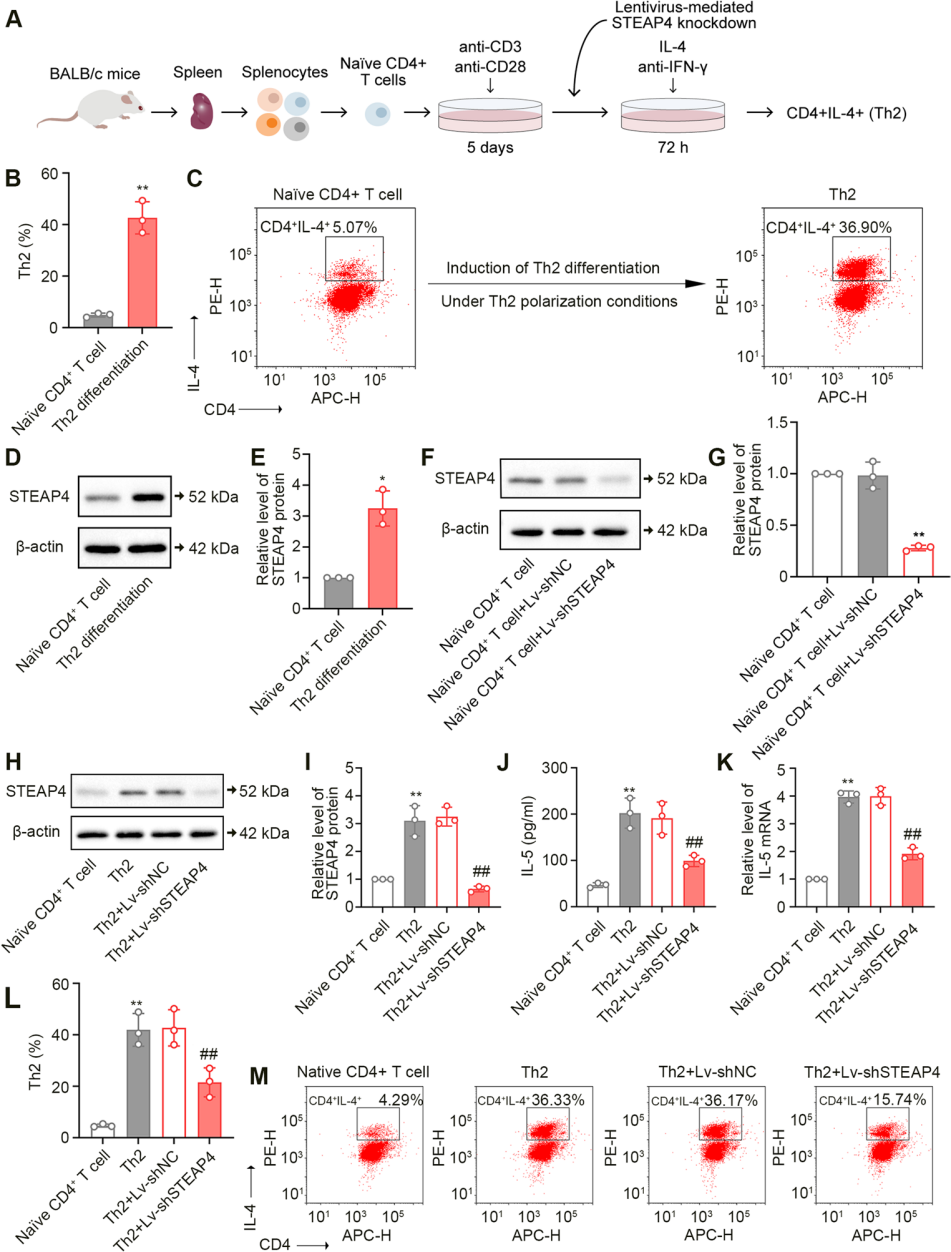
STEAP4 knockdown inhibited Th2 differentiation of naïve CD4^+^ T cells in vitro. (**A**) The schematic diagram of naïve CD4^+^ T cell isolation and Th2 differentiation. (**B**) The percentage of Th2 cells in naïve CD4^+^ T cells under Th2 polarization conditions (*n* = 3). (**C**) Scatter plots for the percentage of Th2 cells in naïve CD4^+^ T cells under Th2 polarization conditions. (**D**) The protein expression of STEAP4 in naïve CD4^+^ T cells under Th2 polarization conditions. (**E**) Quantitative analysis of the STEAP4 expression in Fig. 6D (*n* = 3). (**F**) The protein expression of STEAP4 in naïve CD4^+^ T cells with STEAP4 knockdown. (**G**) Quantitative analysis of the STEAP4 expression in Fig. 6F (*n* = 3). (**H**) The protein expression of STEAP4 in naïve CD4^+^ T cells with STEAP4 knockdown under Th2 polarization conditions. (**I**) Quantitative analysis of the STEAP4 expression in Fig. 6H (*n* = 3). (**J**) The concentration of IL-5 in supernatant of naïve CD4^+^ T cells with STEAP4 knockdown under Th2 polarization conditions (*n* = 3). (**K**) The mRNA expression of IL-5 in naïve CD4^+^ T cells with STEAP4 knockdown under Th2 polarization conditions (*n* = 3). (**L**) The percentage of Th2 cells in naïve CD4^+^ T cells with STEAP4 knockdown under Th2 polarization conditions (*n* = 3). (**M**) Scatter plots for the percentage of Th2 cells in naïve CD4^+^ T cells with STEAP4 knockdown under Th2 polarization conditions. For Fig. 6B and E, the Th2 differentiation group vs. the naïve CD4^+^ T cell group, *p < 0.05, **p < 0.01. For Fig. 6G, the naïve CD4^+^ T cell + Lv-shSTEAP4 group vs. the naïve CD4^+^ T cell + Lv-shNC group, **p < 0.01. For Fig. 6I and L, the Th2 group vs. the naïve CD4^+^ T cell group, **p < 0.01; the Th2 + Lv-shSTEAP4 vs. the Th2 + Lv-shNC group, ##*p* < 0.01. Data were presented as the mean ± SD and analyzed by Welch’s t test or one-way ANOVA. Each experiment was independently reproduced

We have found a significant increase in the percentage of Th2 cells following OVA challenge. To verify the role of STEAP4 in Th2 cell differentiation, we freshly purified naïve CD4^+^ T cells from splenocytes of healthy mice and subsequently induced their differentiation into Th2 cells under specific polarization conditions. A schematic representation of this Th2 differentiation process is depicted in Fig. 6A. The markedly increased percentage of Th2 cells confirmed the successful induction of Th2 cell differentiation (Fig. 6B and C). Th2 cells exhibited a higher STEAP4 protein expression compared to naïve CD4^+^ T cells (Fig. 6D and E). Subsequently, STEAP4 knockdown in naïve CD4^+^ T cells was mediated by lentivirus infection. The decreased STEAP4 expression confirmed the successful knockdown of STEAP4 in cells (Fig. 6F and G). Following lentivirus infection, naïve CD4^+^ T cells were subjected to Th2 polarization conditions, and we observed the decreased STEAP4 protein expression in Th2 cells with STEAP4 knockdown (Fig. 6H and I). We next measured the concentration of Th2-related pro-inflammatory cytokine IL-5 in the supernatant of the culture medium and found that STEAP4 knockdown led to a significant reduction in IL-5 secretion (Fig. 6J). Similarly, the IL-5 mRNA expression in Th2 cells was decreased following STEAP4 knockdown (Fig. 6K). Importantly, STEAP4 knockdown inhibited the differentiation of naïve CD4^+^ T cells into Th2 cells (Fig. 6L and M). These results indicated that STEAP4 knockdown inhibited Th2 differentiation.

Additionally, to determine whether STEAP4 expression varies among Th1, Th17, and Treg cells, we isolated CD4^+^ T cells and then cultured them under different polarization conditions (Figure S4A). Our findings revealed that the expression of STEAP4 had no significant changes in Th1, Th17, or Treg cells compared to naïve CD4^+^ cells (Figure S4B-M). These results indicated that the upregulation of STEAP4 expression is specific to Th2 cells.

### GATA3 Transcriptionally Upregulated the STEAP4 Expression

**Fig. 7:**
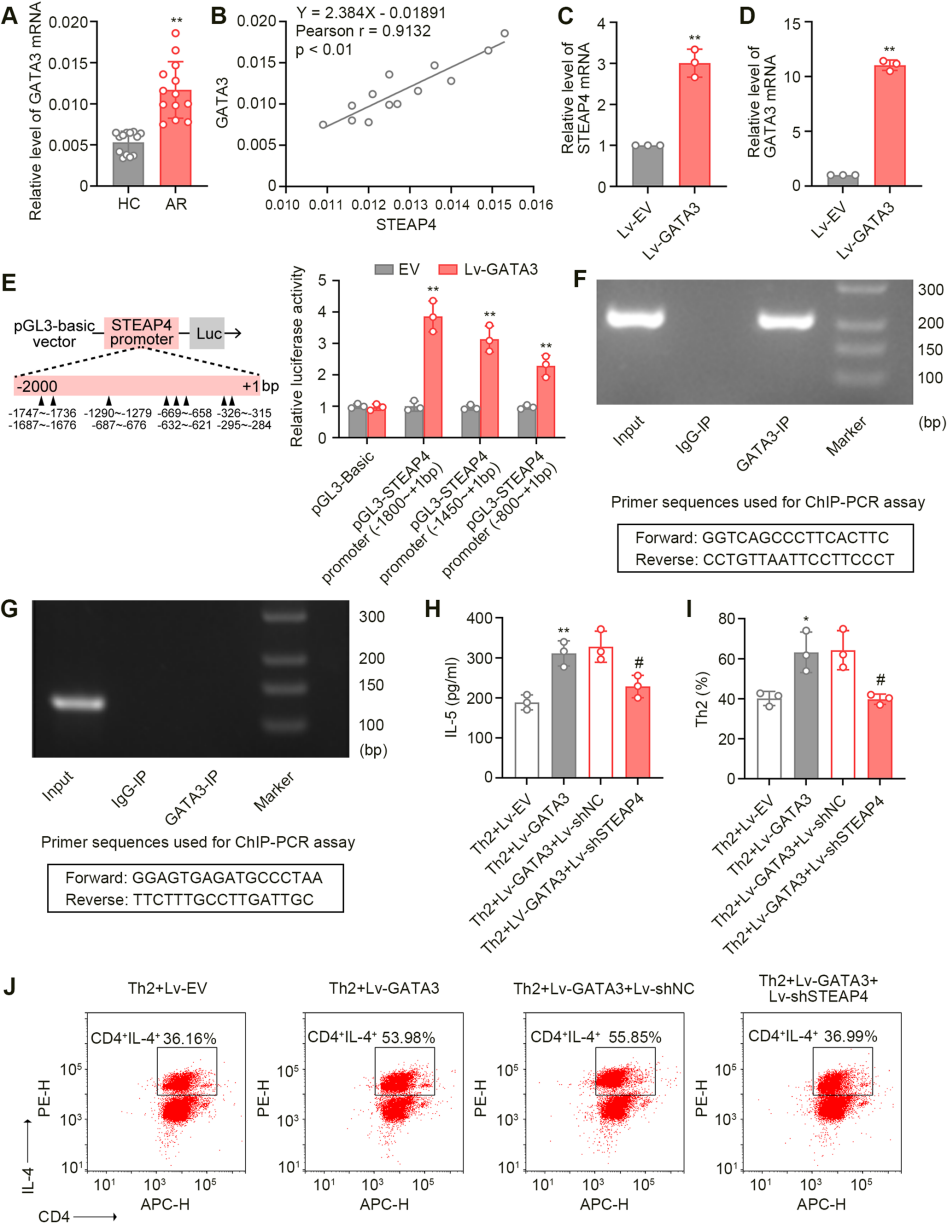
STEAP4 was regulated by GATA3 and mediated the effects of GATA3 on AR. (**A**) The mRNA expression of GATA3 in nasal mucous tissues of patients (*n* = 13). (**B**) Pearson correlation of the mRNA expression between STEAP4 and GATA3 in nasal mucous tissues of patients. (**C**) The mRNA expression of STEAP4 in naïve CD4^+^ T cells with GATA3 overexpression (*n* = 3). (**D**) The mRNA expression of GATA3 in naïve CD4^+^ T cells with GATA3 overexpression (*n* = 3). (**E**) The schematic diagram of the GATA3 binding to the STEAP4 promoter predicted from the JASPAR database, and the relative luciferase activity of the pGL3-basic construct containing the STEAP4 promoter (*n* = 3). (**F**) ChIP-PCR analysis for the interaction between GATA3 and the STEAP4 promoter using the primers to amplify the promoter region of STEAP4 that contains the GATA3 binding site. (**G**) ChIP-PCR analysis for the interaction between GATA3 and the STEAP4 promoter using the primers to amplify the promoter region of STEAP4 without the GATA3 binding site. (**H**) The concentration of IL-5 in supernatant of naïve CD4^+^ T cells with GATA3 overexpression and STEAP4 knockdown under the Th2 polarization condition (*n* = 3). (**I**) The percentage of Th2 cells in naïve CD4^+^ T cells with GATA3 overexpression and STEAP4 knockdown under the Th2 polarization condition (*n* = 3). (**J**) Scatter plots for the percentage of Th2 cells in naïve CD4^+^ T cells with GATA3 overexpression and STEAP4 knockdown under the Th2 polarization condition. For Fig. 7A, the AR group vs. the HC group, **p < 0.01. For 7 C-7E, the Lv-GATA3 group vs. the Lv-EV group, **p < 0.01. For 7 H and 7I, the Th2 + Lv-GATA3 group vs. the Th2 + Lv-EV group, *p < 0.05, **p < 0.01; the Th2 + Lv-GATA3 + Lv-shSTEAP4 group vs. the Th2 + Lv-GATA3 + Lv-shNC group, #*p* < 0.05. Data were presented as the mean ± SD and analyzed by Mann-Whitney test, simple linear regression analysis, two-way ANOVA, Welch’s t test, or one-way ANOVA. Each experiment was independently reproduced

GATA3 is a critical transcription factor involved in Th2 differentiation. In this study, we predicted the binding sites of GATA3 on the promoter region of STEAP4 using an online database. To investigate the regulatory relationship between GATA3 and STEAP4 in AR, we first analyzed the the GATA3 mRNA expression in clinical samples and found a significant increase in AR compared to healthy controls (Fig. 7A). Subsequently, Pearson correlation analysis was performed to assess the relation between STEAP4 and GATA3 expression levels, revealing a positive correlation (Fig. 7B). For further evaluation, purified naïve CD4^+^ T cells were infected with lentivirus expressing GATA3 or the corresponding empty vector. Following Real-time PCR revealed that GATA3 overexpression significantly increased STEAP4 mRNA levels in these cells (Fig. 7C), with successful GATA3 overexpression confirmed by elevated GATA3 mRNA (Fig. 7D). We examined the timing of this effect by infecting cells with Lv-GATA3 and measuring mRNA expression at 4, 24, and 48 h post-infection. GATA3 overexpression was effectively achieved at 24 and 48 h, coinciding with significantly higher STEAP4 expression. However, no effects were observed at 4 h, indicating a time-dependent regulation of STEAP4 by GATA3 (Fig. S5A). Importantly, STEAP4 knockdown did not affect GATA3 expression, highlighting the specificity of this regulatory pathway (Fig. S5B). The JASPAR website predicted several binding sites of GATA3 in the STEAP4 promoter region. Next, a dual luciferase reporter assay was performed to examine GATA3 binding to various regions of the STEAP4 promoter (−1800 ~ + 1 bp, −1450 ~ + 1 bp, and − 800 ~ + 1 bp) in HEK293T cells. The results showed that GATA3 bound to the STEAP4 promoter, as evidenced by a significant increase in luciferase activity across all tested regions upon GATA3 overexpression (Fig. 7E). Further validation was conducted through PCR analysis of the ChIP assay, utilizing primers to amplify the promoter region of STEAP4 that contains the GATA3 binding site. The results confirmed the binding of GATA3 to the DNA region of STEAP4 (Fig. 7F). In contrast, primers targeting a region lacking the binding site demonstrated no interaction (Fig. 7G). Additionally, GATA3 overexpression also elevated the Th2 cell percentage and the IL-5 production, but STEAP4 knockdown reversed these effects (Fig. 7H and J). These findings indicated that GATA3 regulated STEAP4 expression and contributed to the development of AR.

## Discussion

Th2 cells-mediated immune response is responsible for the pathogenesis of most allergic diseases [30]. Mechanistically, allergen-sensitized Th2 cells produce pro-inflammatory cytokines such as IL-4, IL-5, and IL-13, thereby driving airway eosinophilia and chronic inflammation [31]. Activated Th2 cells inhibit the activity of Th1 cells, leading to an imbalance of Th1/Th2 cells [29]. Consequently, the restoration of this Th1/Th2 imbalance has been acknowledged as a critical factor in mediating anti-allergic effects in allergic diseases [32]. Th2 differentiation of CD4^+^ T cells is a characteristic feature of AR [6]. Therefore, we aimed to explore potential targets that affect Th2 differentiation, with the intention of providing novel insights into the treatment of AR. Our results suggested a decreased percentage of Th1 cells and an increased percentage of Th2 cells in the spleens of OVA-challenged mice. The spleen, as the largest secondary lymphoid organ, reflects the current immune status [33]. Moreover, previous high-quality studies have utilized the spleen to assess the proportion of Th2 cells in the in vivo model of AR [17, 33]. Notably, this study primarily focused on examining the effects of STEAP4 on Th2 cell differentiation under the physiological conditions of AR. For this reason, we isolated splenic CD4^+^ T cells for in vivo differentiation studies. Additionally, Th1 cells induce the production of IgG2a antibody, while Th2 cells stimulate the production of IgG1 and IgE antibodies [34]. Our results detected a reduction in IgG2a accompanied by an elevation in IgG1 and IgE antibodies in the AR mouse model, confirming a Th1/Th2 imbalance. In this study, we explored the role of STEAP4 in maintaining the Th1/Th2 balance and its influence on Th2 differentiation. Our results suggest that STEAP4, regulated transcriptionally by GATA3, affects Th2 differentiation and contributes to the development of AR.

STEAP4 consists of an N-terminal cytoplasmic oxidoreductase domain and a six-helical transmembrane domain [35]. Prior research has evidenced its role in inflammatory diseases. The pro-inflammatory effects of STEAP4 have been identified in Th17-induced autoimmune encephalomyelitis and intestinal inflammation [10, 11]. Conversely, it has also been recognized as an anti-inflammatory protein in response to pro-inflammatory cytokines like TNF-α and IL-6 [36]. Moreover, STEAP4 exerted an inhibitory effect on TNF-α-induced rheumatoid arthritis [37]. These previous studies collectively suggest a dual role of STEAP4 in inflammation. However, its exact role in the development of the upper airway inflammatory disease AR has not been elucidated yet. We found the increased STEAP4 expression in the nasal mucous tissues of patients with AR and OVA-challenged mice with AR. Notably, most studies have used 6–8-week-old mice to model AR. However, AR affects all age groups [38]. Therefore, the present study referenced a previously published report and chose 4-week-old mice for the experiments [14].

Following the implementation of loss of STEAP4 function experiments in vivo, we determined the anti-inflammatory property of STEAP4 knockdown on inflammatory mediators (such as histamine and inflammatory cytokines) and inflammatory cell infiltration in NALF under the pathological condition of AR. Histamine is a key mediator of allergic responses primarily released by mast cells and basophils, and an increase in histamine levels indicate activation of these cells [3]. In our study, we observed a decrease in serum histamine levels in OVA-treated mice with STEAP4 knockdown, suggesting a potential decrease in the activity of mast cells and/or basophils. To assess inflammatory cell infiltration in the NALF, we employed Diff-Quik staining. Usually, flow cytometry typically provides greater accuracy for identifying cell types. What’s more, phenotyping M1 or M2 macrophages in the NALF of mice using flow cytometry contributes to further supporting the inflammatory state in the nasal environment of mice with AR. However, the number of cells in the NALF of mice is relatively limited, particularly when the mice are in a normal physiological state or have low levels of inflammation. Flow cytometry requires a larger number of cells for effective detection. The purpose of this experiment is to assess changes in the number of inflammatory cells in the NALF of mice following an OVA challenge or an OVA challenge combined with STEAP4 knockdown. This could be effectively achieved using Diff-Quik staining. Additionally, research on AR often counts inflammatory cells in mouse NALF using staining techniques, though flow cytometry is less commonly employed. Therefore, in this study, we counted the number of inflammatory cells through staining in accordance with established research practices [39, 40]. Given the Th1/Th2 imbalance implicated in the pathogenesis of AR, we tried to explore whether the underlying mechanism of STEAP4 knockdown in AR was associated with the change of Th1/Th2 balance. Consistently, STEAP4 knockdown reversed the percentages of Th1 and Th2 cells, the corresponding cytokines, and specific serum antibodies in vivo, and inhibited Th2 differentiation in vitro, suggesting its regulatory role in the Th1/Th2 imbalance by inhibiting Th2 cells. Furthermore, STEAP4 knockdown was found to inhibit the insulin-stimulated AKT activation [41]. Huang et al. suggested that OVA-induced Th2 differentiation was associated with the AKT activation [42]. These prior studies suggest that the regulatory mechanism of STEAP4 in Th2 differentiation might be linked to the AKT signaling pathway.

GATA3 is a transcription factor that plays a crucial role in Th2 differentiation [43]. Research has shown that allergen challenges led to increased GATA3 expression in the upper airway mucosa of patients suffering from AR [13], indicating a strong link between GATA3 and the development of AR. Moreover, GATA3 overexpression has been shown to exacerbate allergen-induced airway inflammation in mice [44]. This prompted us to investigate whether GATA3 acts as an upstream regulator of STEAP4 in the context of AR. Consistent with the STEAP4 expression pattern, AR samples had a higher level of GATA3 expression compared to control samples. GATA3 overexpression elevated the STEAP4 protein expression in naïve CD4^+^ T cells. Furthermore, GATA3 overexpression enhanced the relative luciferase activity of the STEAP4 promoter region, including the − 1800 ~ + 1 bp, −1450 ~ + 1 bp, and − 800 ~ + 1 bp. We noted that, after gradual truncation of the STEAP4 promoter region, there was still a high luciferase activity in the STEAP4 promoter region of −800 ~ + 1 bp, indicating a higher likelihood of transcription factor binding within this segment. Next, the promoter sequences in this region were selected for subsequent ChIP-PCR experiments, which showed that GATA3 effectively bound to the STEAP4 promoter. These results indicated that GATA3 might be an upstream effector of STEAP4 in the development of AR. The rescue experiments were performed to check whether GATA3 would regulate the STEAP4’s role in Th2 differentiation. The results demonstrated that GATA3 overexpression promoted the Th2 differentiation, which was reversed in part by STEAP4 knockdown, revealing that GATA3 is the upstream molecular effector of STEAP4 under the AR condition. Previous reports have shown that GATA3 directly initiates the transcription of inflammatory factors such as IL-4 and IL-5 [45]. In the present study, we detected the IL-5 concentration in the supernatant of CD4^+^ T cells under the condition of Th2 cell differentiation. Our results showed that GATA3 overexpression increased the production of IL-5 in the supernatant of cell culture, and the role of GATA3 overexpression was rescued by STEAP4 knockdown. It is worth noting that the IL-5 secretion in the Th2 + Lv-GATA3 + Lv-shSTEAP4 group still tended to be slightly higher than that of the Th2 + Lv-EV group, suggesting the possibility that STEAP4 knockdown cannot completely reverse the effect of GATA3 overexpression on IL-5 secretion. Therefore, we speculated that GATA3 might directly initiates IL-5 transcription, or regulate the transcription of other factors and further indirectly regulate the IL-5 expression.

**Fig. 8:**
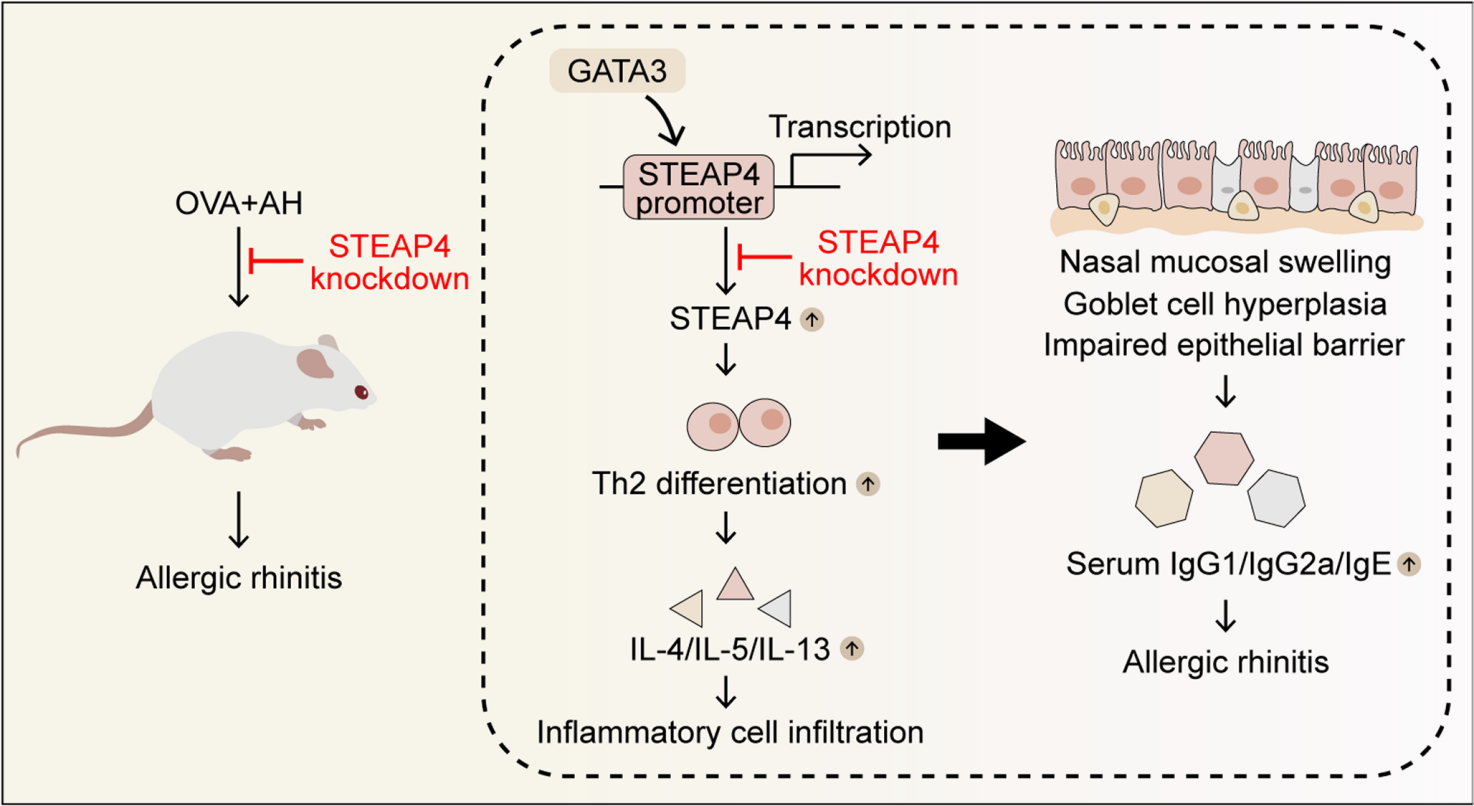
Graphical abstract

Collectively, our study demonstrates that the increased STEAP4 expression, positively regulated by GATA3, is associated with the development of AR. Furthermore, STEAP4 knockdown alleviates AR symptoms by inhibiting Th2 differentiation-mediated inflammation (Fig. 8). While this study contributes insights into potential therapeutic approaches for AR, it also highlights certain limitations. Specifically, the exact mechanisms by which STEAP4 regulates Th2 differentiation remain unclear. Existing research suggested that STEAP4 might regulate Th2 cell differentiation through the AKT signaling pathway and thereby participate in the development of AR. Further investigations will be performed to address this issue in future research.

## Supplementary Information

Below is the link to the electronic supplementary material.

Supplementary Material 1

## Data Availability

Data of this study are available from the corresponding author upon reasonable request.

## Abbreviations

AR: Allergic rhinitis
STEAP4: Six-transmembrane epithelial antigen of prostate 4
GATA3: Guanine adenine thymine adenine sequence-binding protein 3
Lv-shSTEAP4: Lentiviral shRNA targeting STEAP4
Lv-shNC: Lentiviral non-targeting shRNA
OVA: Ovalbumin
IgE: Immunoglobulin E
Th: T helper
STAMP2: Six transmembrane protein of the prostate 2
DSS: Dextran Sulfate Sodium
DEGs: Differentially expressed genes
GO: Gene ontology
BP: Biological process
AH: Aluminum hydroxide
LPC: Lysophosphatidylcholine
cDNA: Complementary DNA
Ct: Cycle threshold
H&E: Hematoxylin and eosin
PAS: Periodic acid-Schiff
NALF: Nasal lavage fluid
PMA: Phorbol12-myristate13-acetate
