# Supplementary material for: The GATA3-STEAP4 Axis Drives Inflammation by Promoting Th2 Differentiation in Allergic Rhinitis
Source: Inflammation. 2025 Dec 20;49(1):13. doi: 10.1007/s10753-025-02381-7 (PMC12722505; doi:10.1007/s10753-025-02381-7)
Supplement: Supplementary file 1 — Supplementary Material 1 [file 10753_2025_2381_MOESM1_ESM.docx]

*Inflammation*

**The GATA3-STEAP4 axis drives inflammation by promoting Th2 differentiation in allergic rhinitis**

Xiaoxu Ding#; Hui Su#; Tiancong Liu#; Yu Chen; Zhao Gao; Ziwen Chang; Weiliang Bai*

Department of Otolaryngology Head and Neck Surgery, Shengjing Hospital of China Medical University, Shenyang, China

#These authors contributed equally to this work

*Corresponding author: Weiliang Bai

E-mail: bweiliangcmu@163.com

**Table S1** Sequences of specific primers for genes

| **Gene** | **Sequences** |
| --- | --- |
| homo GATA3 | F: CGTCCTGTGCGAACTGTCA  R: TCTGGATGCCTTCCTTCTTC |
| homo STEAP4 | F: GATGGAGATTGGGAAAC  R: CAGATGGCAAAGAAGTGA |
| homo β-actin | F: TCAGGGTGAGGATGCCTCTC  R: CTCGTCGTCGACAACGGCT |
| mus GATA3 | F: GCCAGGCAAGATGAGAAA  R: GGTGGGAAGAGTCCAGAGC |
| mus IL-5 | F: GGCTTCCTGTCCCTACT  R: CTTCCATTGCCCACTCT |
| mus IL-13 | F: TTGCCTTGGTGGTCTCG  R: CAATATCCTCTGGGTCCTGT |
| mus occludin | F: CCTGGAGGTACTGGTCT  R: ATCTTTCTTCGGGTTTT |
| mus STEAP4 | F: CCCACTGGACCAAGGAT  R: AGAAGACGCACAGCACA |
| mus IFNγ | F: AGTGGCATAGATGTGGAA  R: CTCAAACTTGGCAATACTC |
| mus IL-4 | F: CATCCTGCTCTTCTTTCTC  R: TTCTCCTGTGACCTCGTT |
| mus ZO-1 | F: ATGAGCGGGCTACCTTA  R: GGAGACTGCGTGGAATG |
| mus β-actin | F: GCCAGAGCAGTAATCTCCTTCT  R: AGTGTGACGTTGACATCCGTA |

**
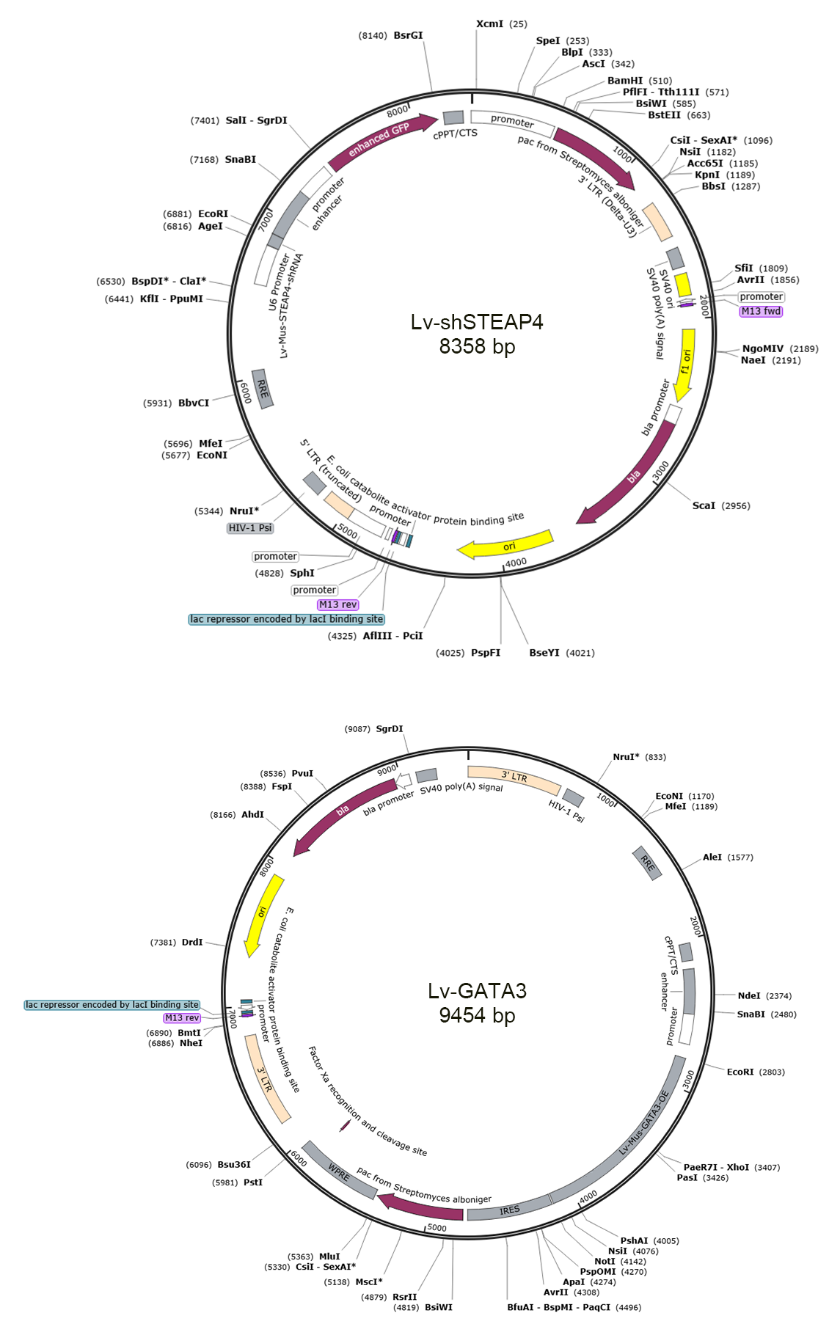
**

**Fig. S1** The maps of recombinant shuttle vectors

**
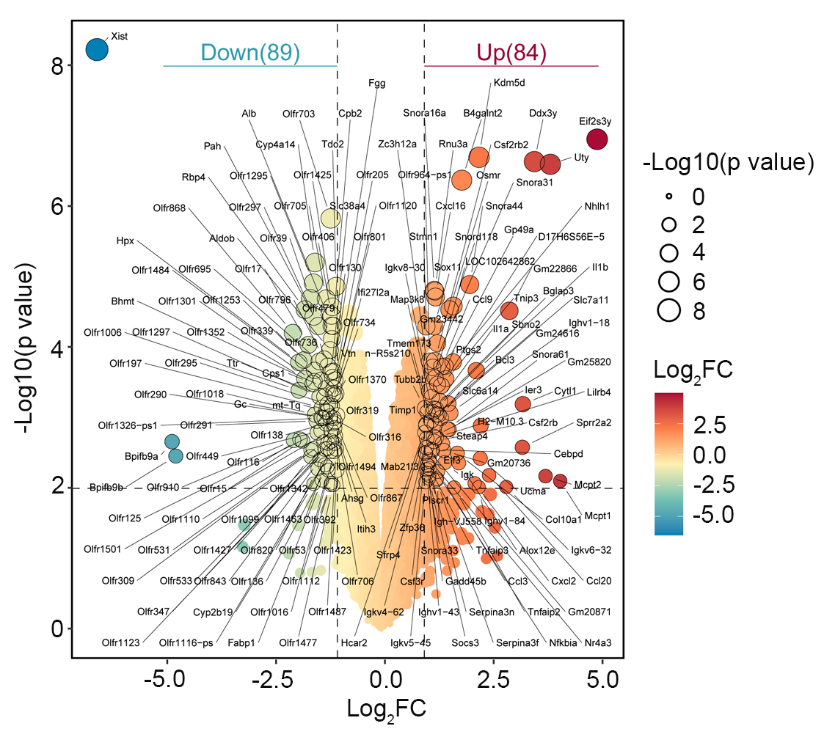
**

**Fig. S2** The volcano plot of identified DEGs in GSE52804

**
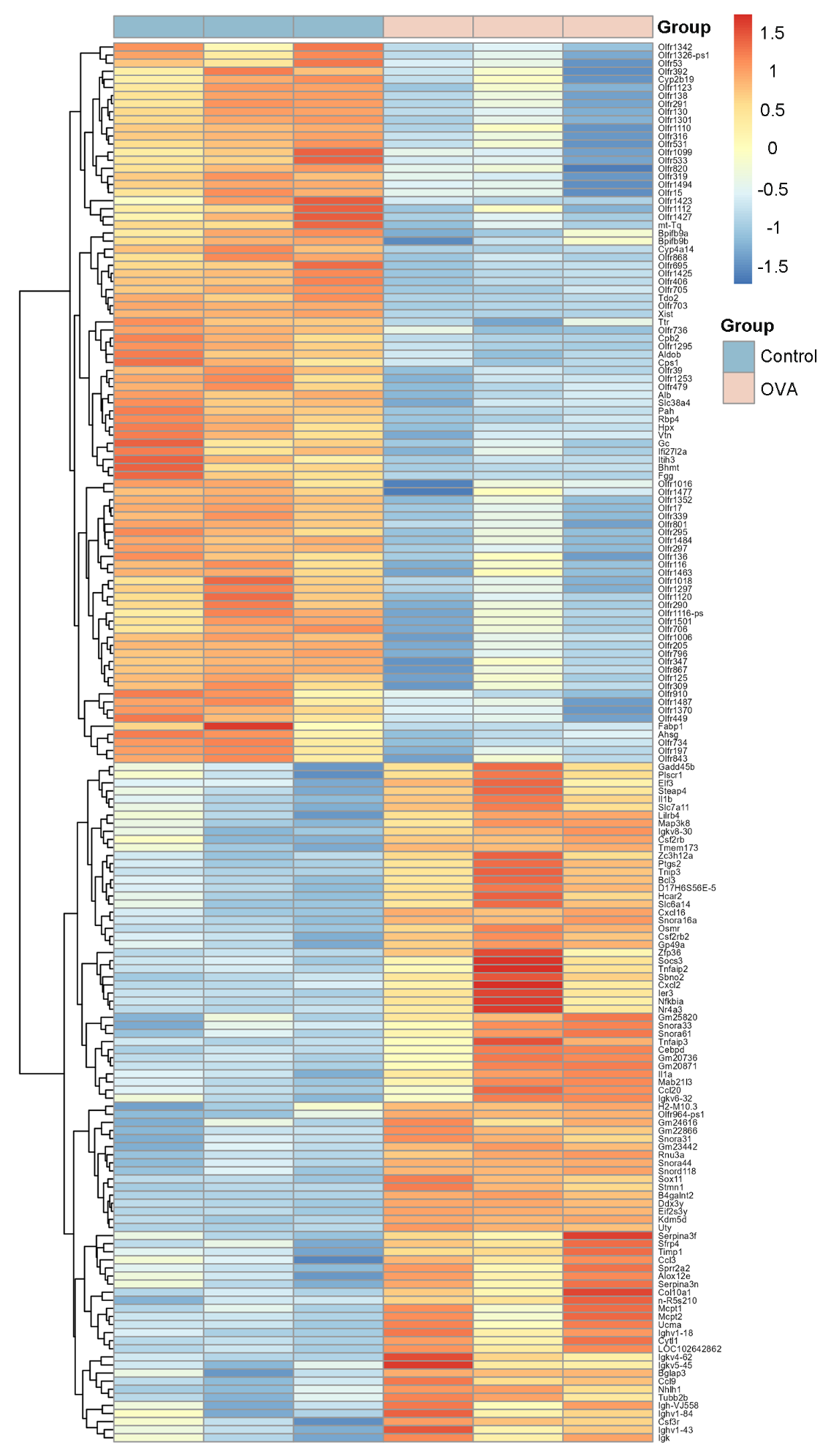
**

**Fig. S3** The heat map of identified DEGs in GSE52804

**
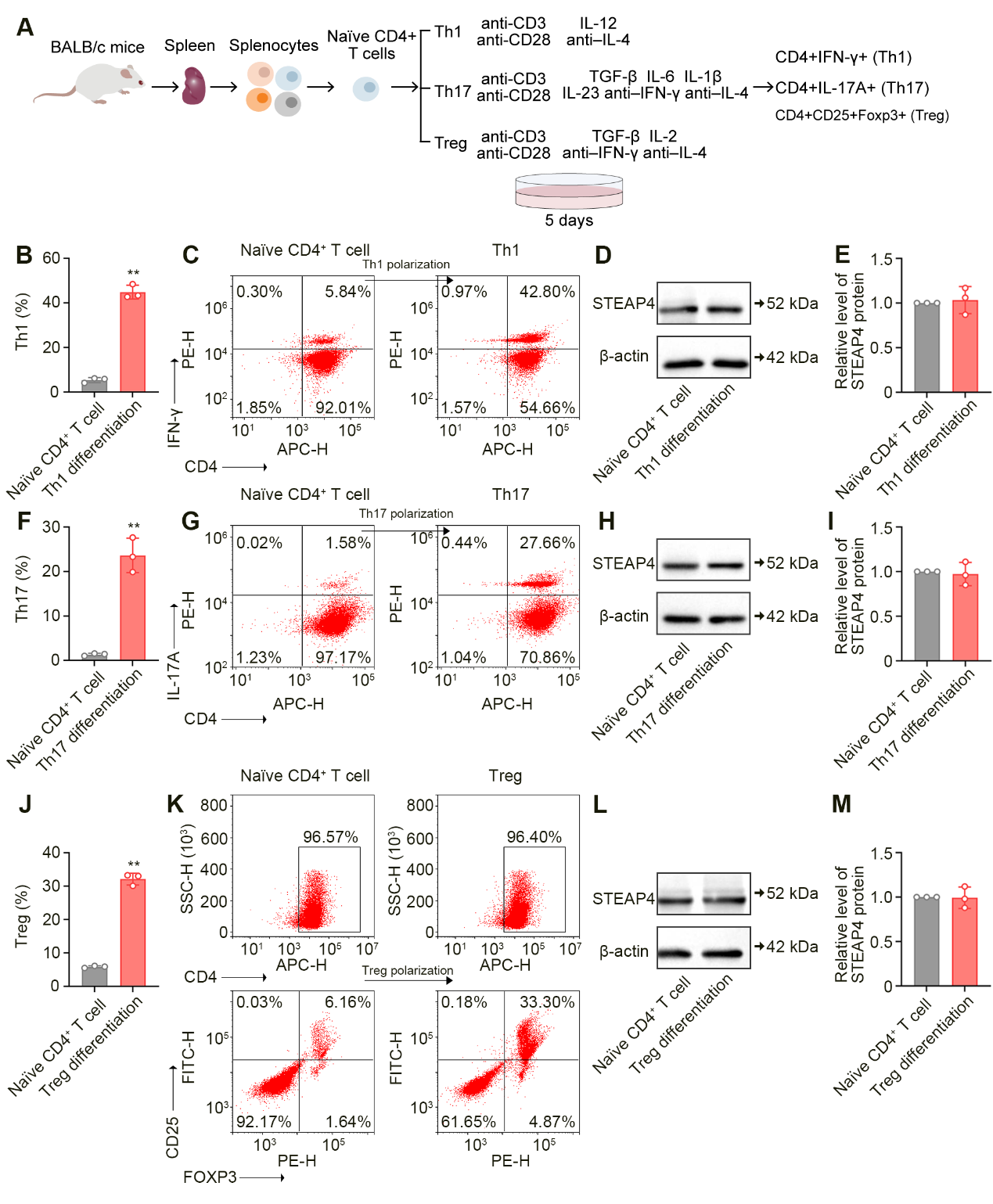
**

**Fig. S4** The expression of STEAP4 in naïve CD4^+^ T cells under Th1, Th17, Treg polarization conditions

(A) The schematic diagram of naïve CD4^+^ T cell isolation and Th1, Th17, or Treg differentiation. (B) The percentage of Th1 cells in naïve CD4^+^ T cells under Th1 polarization conditions (n = 3). (C) Scatter plots for the percentage of Th1 cells in naïve CD4^+^ T cells under Th1 polarization conditions. (D) The protein expression of STEAP4 in naïve CD4^+^ T cells under Th1 polarization conditions. (E) Quantitative analysis of the STEAP4 expression in Fig. S4D (n = 3). (F) The percentage of Th1 cells in naïve CD4^+^ T cells under Th17 polarization conditions (n = 3). (G) Scatter plots for the percentage of Th17 cells in naïve CD4^+^ T cells under Th17 polarization conditions. (H) The protein expression of STEAP4 in naïve CD4^+^ T cells under Th17 polarization conditions. (I) Quantitative analysis of the STEAP4 expression in Fig. S4H (n = 3). (J) The percentage of Th1 cells in naïve CD4^+^ T cells under Treg polarization conditions (n = 3). (K) Scatter plots for the percentage of Treg cells in naïve CD4^+^ T cells under Treg polarization conditions. (L) The protein expression of STEAP4 in naïve CD4^+^ T cells under Treg polarization conditions. (M) Quantitative analysis of the STEAP4 expression in Fig. S4L (n = 3). For Fig. S4B and S4E, the Th1 differentiation group vs. the naïve CD4^+^ T cell group, **p < 0.01. For Fig. S4F and S4I, the Th17 differentiation group vs. the naïve CD4^+^ T cell group, **p < 0.01. For Fig. S4J and S4M, the Treg differentiation group vs. the naïve CD4^+^ T cell group, **p < 0.01. Data were presented as the mean ± SD and analyzed by unpaired t test or Welch's t test. Each experiment was independently reproduced.

**
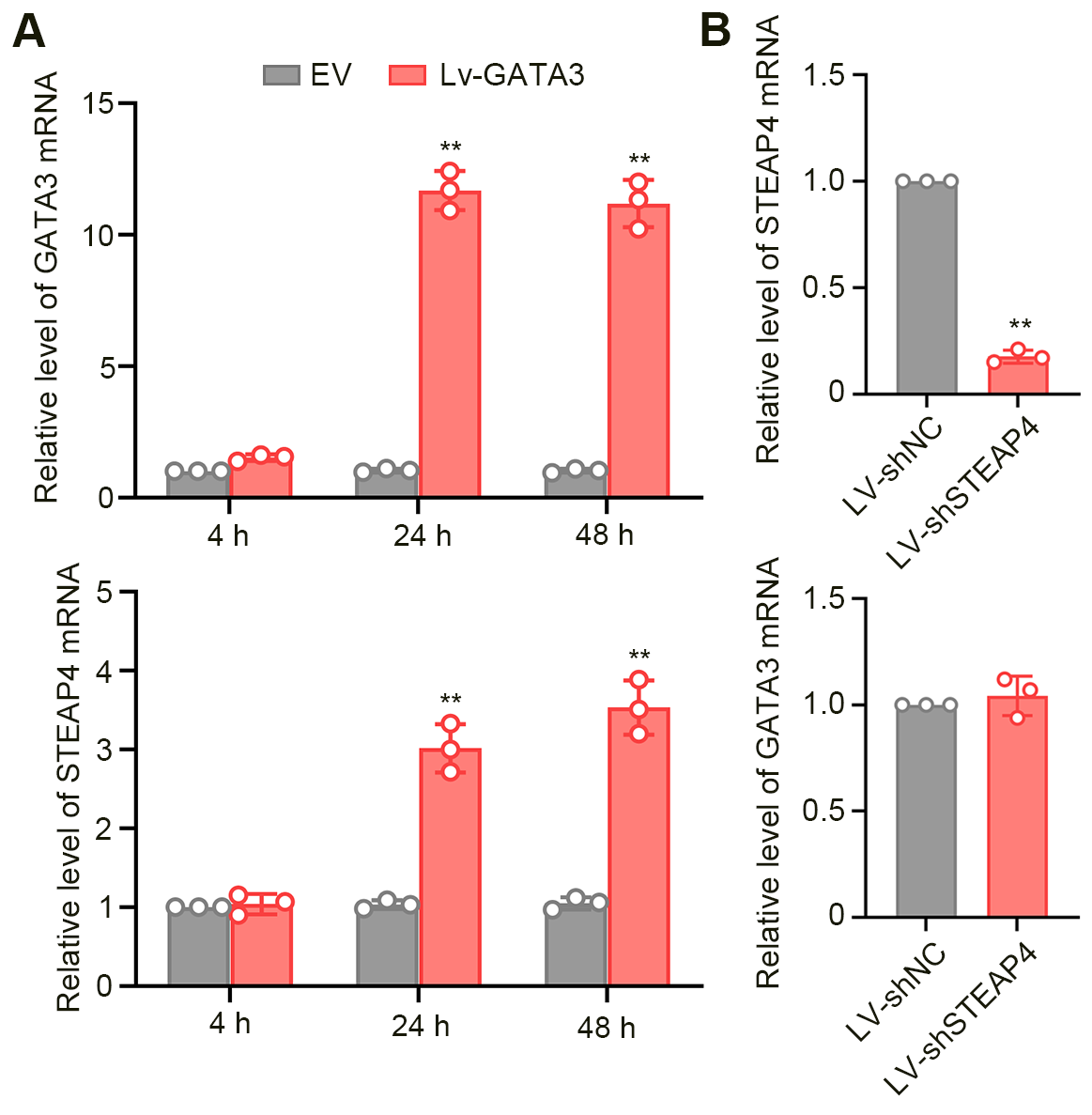
**

**Fig. S5** The regulatory relationship between GATA3 and STEAP4

(A) The mRNA expression of GATA3 and STEAP4 in naïve CD4^+^ T cells infected with EV or Lv-GATA3 for 4, 24, or 48 h (n = 3). (B) The mRNA expression of STEAP4 and GATA3 in naïve CD4^+^ T cells with STEAP4 knockdown (n = 3). For Fig. S5A, the Lv-GATA3 group vs. the EV group, **p < 0.01. For S5B, the Lv-shSTEAP4 group vs. the Lv-shNC group, **p < 0.01. Data were presented as the mean ± SD and analyzed by two-way ANOVA or Welch's t test. Each experiment was independently reproduced.
